# Haem-activated promiscuous targeting of artemisinin in *Plasmodium falciparum*

**DOI:** 10.1038/ncomms10111

**Published:** 2015-12-22

**Authors:** Jigang Wang, Chong-Jing Zhang, Wan Ni Chia, Cheryl C. Y. Loh, Zhengjun Li, Yew Mun Lee, Yingke He, Li-Xia Yuan, Teck Kwang Lim, Min Liu, Chin Xia Liew, Yan Quan Lee, Jianbin Zhang, Nianci Lu, Chwee Teck Lim, Zi-Chun Hua, Bin Liu, Han-Ming Shen, Kevin S. W. Tan, Qingsong Lin

**Affiliations:** 1 Department of Biological Sciences, National University of Singapore, Singapore 117543, Singapore; 2 The State Key Laboratory of Pharmaceutical Biotechnology, College of Life Sciences, Nanjing University, Nanjing 210023, China; 3 Interdisciplinary Research Group in Infectious Diseases, Singapore-MIT Alliance for Research & Technology (SMART), Singapore 138602, Singapore; 4 Department of Physiology, Yong Loo Lin School of Medicine, National University of Singapore, Singapore 117597, Singapore; 5 Department of Chemical and Biomolecular Engineering, National University of Singapore, Singapore 117585, Singapore; 6 Department of Microbiology and Immunology, Yong Loo Lin School of Medicine, National University of Singapore, Singapore 117545, Singapore; 7 NUS Environmental Research Institute, Singapore 117411, Singapore; 8 Department of Anaesthesiology, Singapore General Hospital, Singapore 169608, Singapore; 9 School of Traditional Chinese Medicine, Southern Medical University, Guangzhou 510515, China; 10 College of Life Science and Technology, Beijing University of Chemical Technology, Beijing 100029, China; 11 Department of Biomedical Engineering, National University of Singapore, Singapore 117583, Singapore; 12 Department of Mechanical Engineering, National University of Singapore, Singapore 117575, Singapore

## Abstract

The mechanism of action of artemisinin and its derivatives, the most potent of the anti-malarial drugs, is not completely understood. Here we present an unbiased chemical proteomics analysis to directly explore this mechanism in *Plasmodium falciparum*. We use an alkyne-tagged artemisinin analogue coupled with biotin to identify 124 artemisinin covalent binding protein targets, many of which are involved in the essential biological processes of the parasite. Such a broad targeting spectrum disrupts the biochemical landscape of the parasite and causes its death. Furthermore, using alkyne-tagged artemisinin coupled with a fluorescent dye to monitor protein binding, we show that haem, rather than free ferrous iron, is predominantly responsible for artemisinin activation. The haem derives primarily from the parasite's haem biosynthesis pathway at the early ring stage and from haemoglobin digestion at the latter stages. Our results support a unifying model to explain the action and specificity of artemisinin in parasite killing.

## Abstract (web summary)

The mechanism of action of artemisinin, an antimalarial drug, is not well understood. Here, the authors use a labelled artemisinin analogue to show that the drug is mainly activated by haem and then binds covalently to over 120 proteins in the malaria parasite, affecting many of its cellular processes.

## Graphical abstract

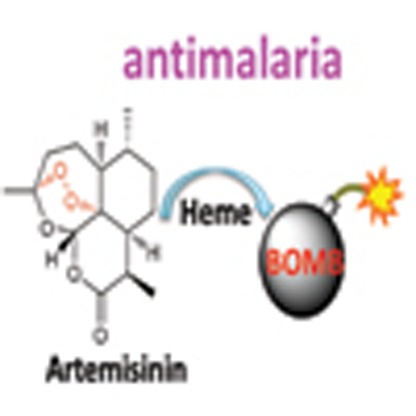

P*lasmodium falciparum*, the most pathogenic human malaria parasite, infects millions of human beings and poses a serious public health threat. Currently, the most potent anti-malarial drugs are artemisinin and its derivatives^1^,^2^. Artemisinin is a sesquiterpene lactone with an endoperoxide bridge^3^. The hallmark of artemisinin activation is the generation of highly reactive carbon-centred radicals via endoperoxide cleavage^4^,^5^,^6^. Even after several decades of intensive studies on the mechanism of action of artemisinin^7^,^8^, two critical yet unresolved long-standing questions remain. The first concerns the origin of iron sources required for artemisinin activation. Both free ferrous iron^9^,^10^ and haem^11^ have been proposed to be the predominant iron sources for its activation, but the results of relevant research are controversial. The second question concerns the exact targets of activated artemisinin. Although several proteins have been reported to be the drug targets^8^,^9^,^12^, none of them could satisfactorily account for the quick and highly potent killing effect of artemisinin. It is possible that activated artemisinin might have many other direct targets due to the promiscuous nature of the radicals^3^,^6^,^8^. To address these questions, it is critical to develop an approach to directly study the artemisinin–protein interaction.

Here we have developed an unbiased chemical proteomics approach and systematically identified 124 artemisinin covalent binding targets. Many of the identified targets are involved in essential biological processes. We have validated several artemisinin–protein interactions through *in vitro* binding assays and orthogonal confirmatory experiments. Importantly, targeted enzyme activity was impaired on artemisinin binding in the tested proteins. Thus, our data support the hypothesis that activated artemisinin may kill the malaria parasite through a promiscuous targeting mechanism. In addition, we use *in vitro* binding analyses to show that haem, rather than free ferrous iron, is predominantly responsible for artemisinin activation. These findings are also supported by an *ex vivo* cancer cell model. The source of haem required for artemisinin is derived from the parasite's haem biosynthesis pathway at the early ring stage and from haemoglobin digestion at latter stages. Thus, the ‘blood-eating' nature of the parasite with the release of extremely high levels of haem confers the high efficacy of artemisinin against the parasites, with minimum side effects towards healthy red blood cells (RBCs). Our results support a unifying model to explain the action and specificity of artemisinin in parasite killing and could facilitate the development of better strategies to treat malaria in times of emerging artemisinin resistance.

## Results

### Synthesis of an artemisinin-based activity probe (AP1)

**Figure 1:**
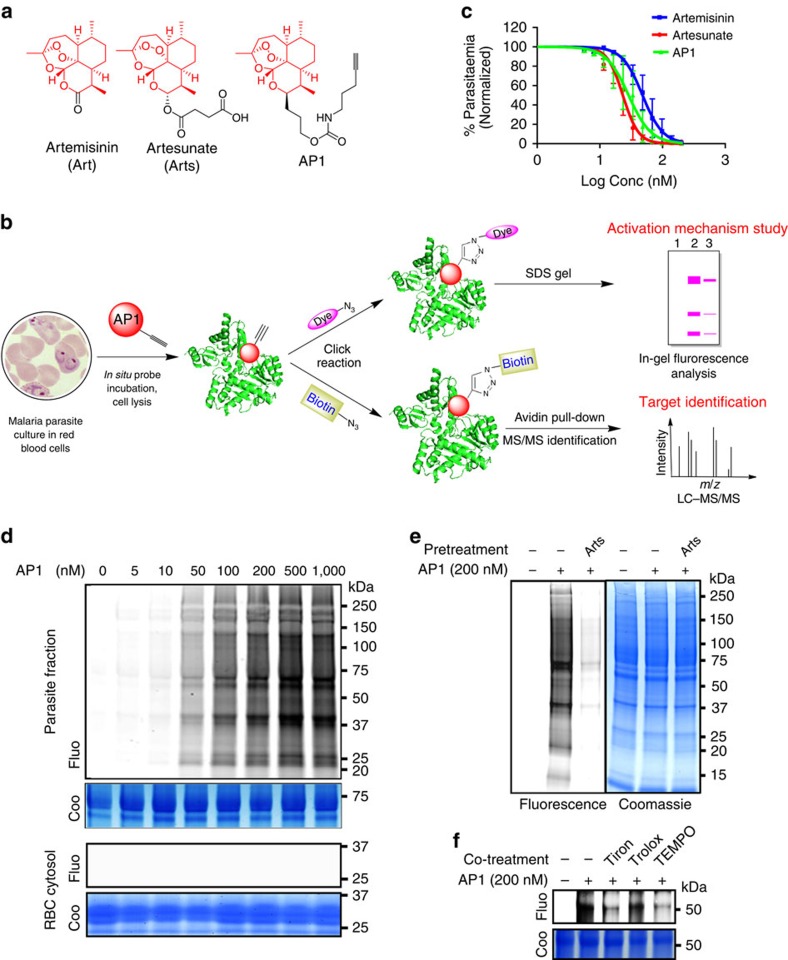
The chemical proteomics approach to study artemisinin's mechanism of action. (**a**) Chemical structures of artemisinin (**Art**), artesunate (**Arts**) and the alkyne-tagged-clickable probe (**AP1**). (**b**) General workflow of the chemical proteomics approach. Fluorescence labelling was used to study the activation mechanism of artemisinin, while biotin pull-downs coupled with LC–MS/MS were used to identify protein targets of artemisinin. (**c**) The killing effect of **AP1** is comparable to that of artemisinin and artesunate on *P. falciparum* 3D7. (**d**) *In situ* parasite labelling with **AP1**. The labelling was dose dependent and specific to parasite proteins. Healthy RBC cytosolic proteins were not labelled. (**e**) The **AP1** *in situ* parasite labelling was artemisinin specific as the excess **Arts** can largely compete with **AP1**-target labelling. (**f**) Free-radical scavenger (Tiron, 1 mM; Trolox, 400 μM; TEMPO, 1 mM) co-treatment reduces the level of parasite protein alkylation by **AP1**. Fluo, fluorescence scanning; Coo, Coomassie staining. Error bars represent s.d. in three independent replicates in **c**. Full-gel images for panels **d** and **f** are shown in Supplementary Fig. 13.

To profile and identify the targets of artemisinin, **AP1**, a chemically engineered artemisinin with a clickable alkyne tag attached^13^,^14^,^15^,^16^,^17^,^18^,^19^,^20^,^21^,^22^, was designed and synthesized (Fig. 1a, probe synthesis scheme is shown in Supplementary Fig. 1). The alkyne tag can be further appended with a fluorescent dye or a biotin moiety through click chemistry, allowing the **AP1** covalent binding targets to be visualized on SDS–polyacrylamide gel electrophoresis (PAGE) or affinity purified for mass spectrometric identification (Fig. 1b).

### Fluorescence labelling of artemisinin targets in parasites

First, we tested the anti-malarial activity of **AP1** on two different parasite strains, the laboratory line *P. falciparum* 3D7 and the field isolate ARS270. **AP1** was as potent as the unmodified artemisinin, suggesting that the addition of the clickable alkyne tag does not interfere with drug activity (Fig. 1c and Supplementary Fig. 2). Crude protein extracts were prepared from **AP1**-treated live parasites, clicked with a fluorescent dye and resolved by SDS–PAGE. Only **AP1** covalent binding targets can be labelled and visualized with fluorescence scanning. Many parasite proteins were fluorescently labelled in an **AP1** dose-dependent manner, whereas **AP1** failed to react with proteins from healthy RBCs under the same conditions (Fig. 1d). This indicates that **AP1** can only be activated inside the parasite but not in the uninfected RBC. We further examined the **AP1** labelling of the infected RBCs. The results showed that some of the host cell proteins were also labelled when the RBCs were infected with parasites, although the labelling level was low (Supplementary Fig. 3). This might have been due to the release of parasite-activated drug into the cytosol of infected RBCs. However, this will not cause significant side effects, as the majority of the activated drug is confined within the infected RBCs, as evidenced by the observation that the drug failed to label the uninfected RBCs. Our results ascertained that artemisinin has high specificity towards the malaria parasite and infected RBCs, with minimum side effects towards healthy RBCs. Moreover, **AP1** labelling was markedly decreased with preincubation of excess artesunate (a water-soluble artemisinin analogue) (Fig. 1e), suggesting that **AP1** binds to the same protein targets as artemisinin. In addition, co-incubation with free-radical scavengers (Tiron, Trolox and TEMPO) also reduced the binding activity of **AP1** (Fig. 1f). Collectively, these results indicate that the engineered **AP1** is pharmacologically similar to artemisinin. The fluorescence intensity of **AP1**-labelled proteins on SDS–PAGE serves as a convenient read-out for **AP1** binding activity.

### Identification of artemisinin targets

Next, we went on to identify the covalent binding targets of **AP1**. To do so, live parasites (unsynchronized) were incubated for 4 h with 500 nM **AP1**, a clinically achievable artemisinin dose^2^,^23^, and subjected to crude protein extract preparation, followed by biotin labelling of the alkyne tag. The **AP1** targets were affinity purified by streptavidin beads and identified with tandem mass spectrometry. A total of 124 parasite proteins were identified as direct targets of artemisinin in three independent experiments and documented in Supplementary Table 1, while the dimethyl sulfoxide (DMSO)-treated control pull-down did not identify any parasite proteins. The statistical analysis and detailed information on the targets are shown in Supplementary Fig. 4 and Supplementary Data 1. Information about the relative abundance of artemisinin targets is shown in Supplementary Fig. 5 and Supplementary Data 1. Among the proteins in our target list, 33 proteins have previously been developed or proposed as anti-malarial drug targets (Supplementary Table 2). These include SERCA/PfATP6, a previously known artemisinin target^9^.

**Figure 2:**
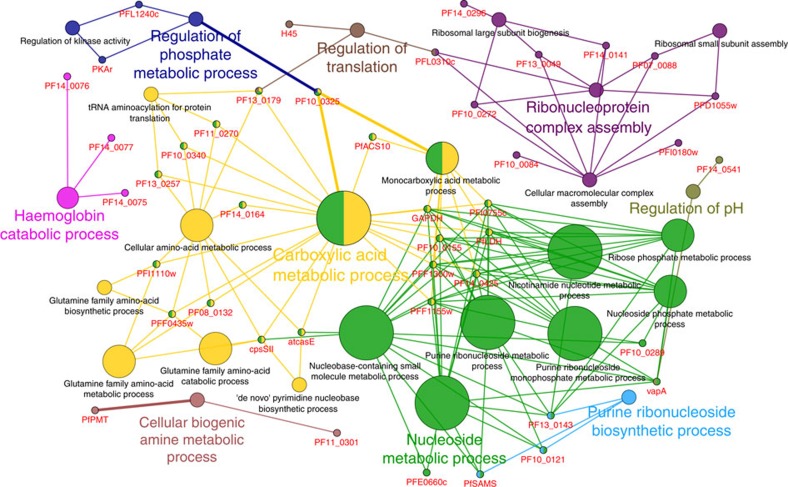
Artemisinin targets are involved in multiple biological processes essential for parasite survival. GO analysis conducted using Cytoscape with Cluego plugin revealed that the **AP1** targets are involved in many essential biological processes of the parasite, including the metabolism of carboxylic acids, cellular biogenic amines and nucleosides, as well as ribonucleoside biosynthesis. This highlights the multi-targeting ability of artemisinin, which exerts numerous effects on the physiological state of the malaria parasite.

We further used pull-downs followed by immunoblotting to validate several selected artemisinin targets. Our results confirmed that **AP1** could successfully pull-down plasmepsinl (PM I), plasmepsin II (PM II), merozoite surface protein 1 (MSP1) and actin (Supplementary Fig. 6). Gene ontology (GO) analysis revealed that the **AP1** targets were involved in many essential biological processes of the parasite, including the carboxylic acid metabolic process, cellular biogenic amine metabolic process, nucleoside metabolic process and ribonucleoside biosynthetic process (Fig. 2). In addition, cellular localization analysis identified several enriched compartments, such as cytosol, vacuolar membrane and notably food vacuoles (Supplementary Fig. 7). Of interest, erythrocyte haemoglobin digestion occurs in the food vacuole^24^ and provides the source of amino acids required to maintain intracellular osmolarity during rapid parasite growth^25^.

**Figure 3:**
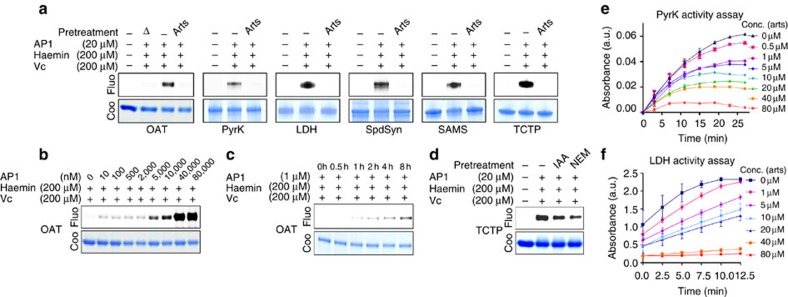
*In vitro* binding and functional validation of artemisinin targets. (**a**) Artemisinin specifically interacts with OAT, PyrK, LDH, SpdSyn, SAMS and TCTP as the unlabelled artesunate (25 ×) can compete with the **AP1** binding. Heat denaturation reduces the **AP1**-labelling level of OAT, suggesting that the interaction of artemisinin with OAT is activity based. (**b**) Dose-dependent labelling of OAT with **AP1** (4 h treatment). (**c**) Time-dependent labelling of OAT with **AP1**. (**d**) The interaction of artemisinin with OAT may involve thiol and amine groups as IAA (blocking thiol, 30 mM) and NEM (blocking amine, 10 mM) pretreatment (20 min) can reduce binding. (**e**,**f**) Activated artesunate inhibits the activities of PyrK (**e**) and LDH (**f**) *in vitro*. Δ, heat denaturation; IAA, iodoacetamide; NEM, *N*-ethylmaleimide; Conc., concentration. Error bars represent s.d. in three independent replicates in **e** and **f**. Full-gel images for panels **a**–**d** are shown in Supplementary Fig. 13.

Several enzymes involved in the key metabolic pathways of the parasite, as revealed in our GO analysis, including ornithine aminotransferase (OAT), pyruvate kinase (PyrK), L-lactate dehydrogenase (LDH), spermidine synthase (SpdSyn) and *S*-adenosylmethionine synthetase (SAMS), were selected to validate their interaction with **AP1** *in vitro* (Table 1). These enzymes were expressed in *Escherichia coli*, purified and incubated with **AP1** in the absence or presence of haem (haemin reduced by L-ascorbic acid (Vc))^26^. Direct interactions between **AP1** and these five enzymes were detected, which were completely thwarted in the presence of excessive amounts of artesunate (Fig. 3a). With OAT as an example, we showed dose- and time-dependent **AP1**–OAT binding (Fig. 3b,c). The **AP1** binding was also protein conformation dependent, as the heat-denatured OAT could not be labelled by **AP1** (Fig. 3a). Notably, OAT has been proposed to be a promising anti-malarial target whose inhibitor has a killing effect at the nano-molar range^27^. In addition, PyrK and LDH are involved in the parasite's glycolytic pathway and are responsible for ATP production. Functional assays showed that artesunate inhibits their enzymatic activities in a dose-dependent manner (Fig. 3e,f). Interestingly, translationally controlled tumour protein (TCTP), a previously identified artemisinin target^12^, was not in our target list. This was likely because TCTP contains many lysine and arginine residues, which resulted in extensive trypsin digestion, making it difficult to identify from the artemisinin target mixture (Supplementary Table 3). Nevertheless, the **AP1**–TCTP interaction was also detected by our *in vitro* assay (Fig. 3a). Furthermore, artemisinin may bind to residues, such as cysteine and lysine, as pretreatment of TCTP with iodoacetamide (IAA) and *N*-ethylmaleimide reduced **AP1** labelling (Fig. 3d).

Overall, we systematically identified the artemisinin targets in *P. falciparum* using our newly developed chemical proteomics approach. With artemisinin binding, the key enzymes, including PyrK and LDH, are covalently modified with the bulky sesquiterpene lactone, which irreversibly disrupts the enzymatic activities. The results indicate that artemisinin may kill the parasite through a promiscuous targeting mechanism.

### Analysis of the activation mechanism of artemisinin

**Figure 4:**
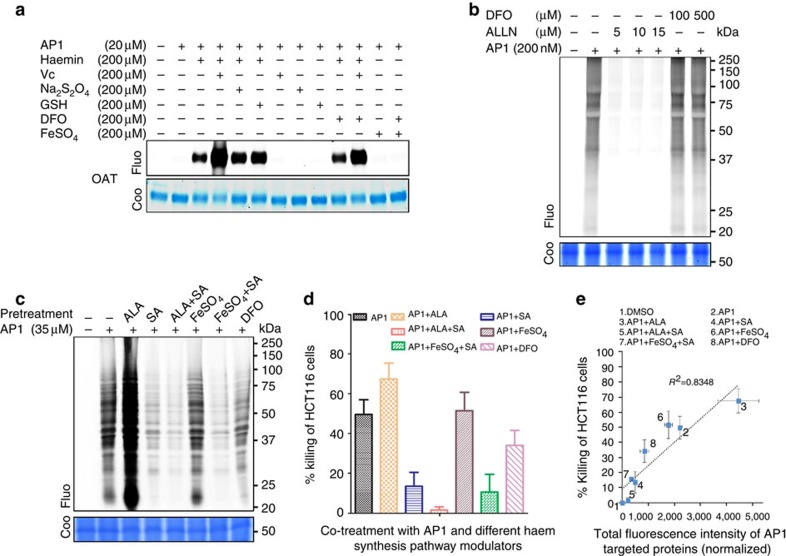
Artemisinin activation is haem dependent. (**a**) Artemisinin's interaction with OAT depends mainly on haemin (200 μM) or haem (200 μM haemin reduced by L-ascorbic acid (Vc, 200 μM), Na_2_S_2_O_4_ (200 μM) or glutathione (GSH, 200 μM)) and much less on ferrous iron (FeSO_4_, 200 μM). Pretreatment (30 min) with DFO (200 μM) slightly reduced the activation of artemisinin. (**b**) For the live parasite, pretreatment (1 h) with the cysteine protease inhibitor ALLN markedly abolished **AP1** labelling of the parasite proteins *in situ*, whereas pretreatment with DFO (30 min) had little effect. (**c**) Modulation of the endogenous haem biosynthesis pathway affects the **AP1** labelling intensity in HCT116 colon cancer cells. Pretreatment (1 h) with the haem synthesis precursor ALA (1 mM) enhances the labelling intensity. Conversely, pretreatment with the haem synthesis inhibitor SA (500 μM) reduces the labelling intensity. ALA and SA co-treatment did not enhance the labelling intensity as SA inhibition occurs downstream of the haem synthesis pathway. Free iron (FeSO_4_, 200 μM) did not affect probe activation. DFO (200 μM) partially reduces the labelling signal intensity, likely due to its role in chelating the free iron, thus impeding the final step of haem synthesis. (**d**) The percentage of HCT116 cells killed by **AP1** treatment or pretreatment with various haem synthesis modulators. (**e**) Correlation between the **AP1** labelling intensity of the total HCT116 target proteins and the percentage of cancer cells killed by **AP1** in the presence of various haem synthesis modulators. Error bars represent s.d. in three independent replicates in **d** and **e**. Full-gel images for panels **a**–**c** are shown in Supplementary Fig. 13.

Our identification of artemisinin targets sheds light on how artemisinin kills the parasite but also provides *in vitro* models to study how artemisinin is activated, particularly whether haem or free ferrous iron is the prerequisite for drug activation. We found that **AP1** itself did not bind to OAT *in vitro* (Fig. 4a). **AP1** binding required the addition of haemin and was further enhanced in the presence of Vc, Na_2_S_2_O_4_ or glutathione (GSH), reagents that reduce haemin to haem (Fig. 4a). By contrast, the addition of ferrous iron had no detectable effect on **AP1**–OAT binding (Fig. 4a). The addition of free-iron chelator deferoxamine (DFO) in the presence of haemin or haem only slightly affected **AP1**–OAT binding (Fig. 4a)^28^.

We therefore reasoned that if haem were indeed the predominant iron source, chelating free ferrous iron should have a minimum effect on **AP1**-target binding *in vivo*. As expected, in live parasites (unsynchronized), **AP1** binding was essentially unaffected in the absence or presence of DFO (up to 500 μM, Fig. 4b) or deferiprone (DFP; up to 500 μM; Supplementary Fig. 8). However, the addition of *N*-acetyl-Leu-Leu-Norleu-al (ALLN), a cysteine protease inhibitor that blocks the digestion of haemoglobin by the parasite to release haem, had a profound inhibitory effect on **AP1** binding (Fig. 4b). This was consistent with previous reports that ALLN treatment antagonizes artemisinin and decreases its activity up to 100-fold^11^,^29^.

Taken together, our results suggest that haem, rather than free ferrous iron, plays a predominant role in artemisinin activation. Previous results have shown that high concentrations of DFP (500 μM) can antagonize the potency of artemisinin by increasing the half-maximal inhibitory concentration (IC_50_) value up to fivefold^10^, and this may be explained by the inhibition of iron-related oxidative stress by chelation and by the protection of oxidative damage via iron-independent pathways^6^,^30^,^31^,^32^,^33^,^34^.

### Activation of artemisinin in an *ex vivo* cancer model

We also utilized cancer cells as an *ex vivo* model to further validate the primary roles of haem in artemisinin activation. It has been reported that cancer cells have enhanced haem biosynthesis and are therefore vulnerable to artemisinin^35^,^36^. As expected, the addition of **AP1** covalently modified many proteins in HCT116 colon cancer cells, leading to cell death (Fig. 4c,d). The addition of a haem precursor, δ-aminolevulinic acid (ALA), significantly increased **AP1** binding, and its binding was blocked in the presence of the haem synthesis inhibitor succinylacetone (SA) (Fig. 4c). Consistent with the above results, free ferrous iron had little effect on **AP1** binding. The partial inhibitory effect of DFO might have been due to the iron chelation that both inhibits iron-related oxidative stress and sequesters the iron required for haem synthesis in cancer cells (Fig. 4c)^6^,^30^,^31^,^32^,^33^,^34^. Importantly, the extent of **AP1** binding in various tested conditions correlated well with its killing effect on cancer cells (Fig. 4e).

### Activation of artemisinin at different parasite stages

**Figure 5:**
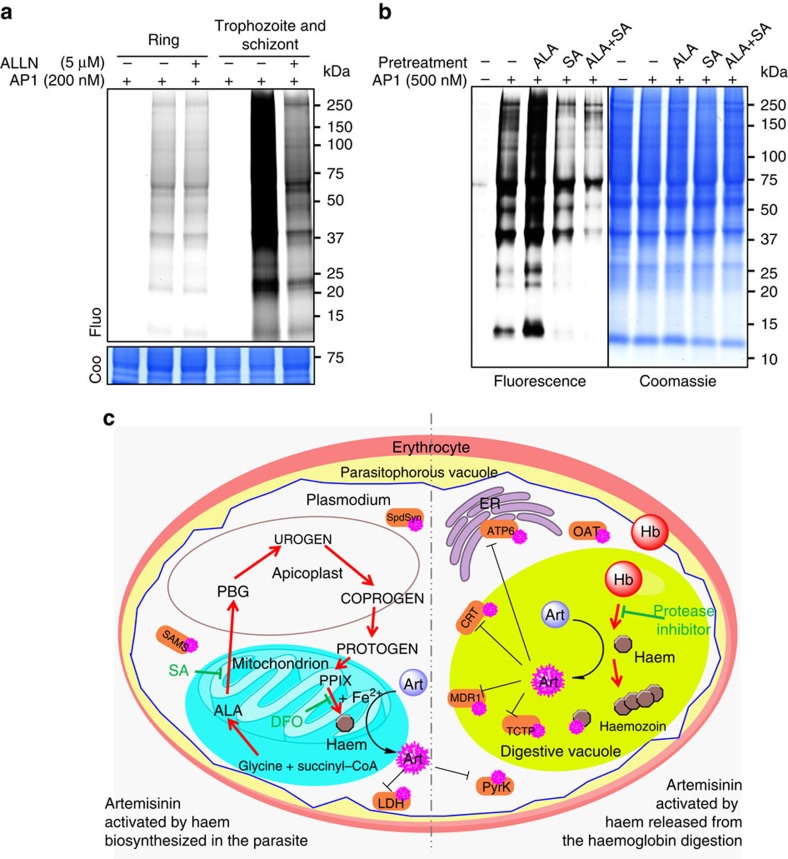
Artemisinin activation in *P. falciparum* relies on two distinct haem sources. (**a**) **AP1** fluorescence-labelling intensity is much lower at the early ring stage compared with the latter stages (trophozoite and schizont stages) of the parasite. The cysteine protease inhibitor ALLN reduced the labelling intensity at the latter stages but could not reduce the labelling intensity at the early ring stage. (**b**) Modulation of the haem biosynthesis pathway of the parasite affected the labelling intensity of **AP1** in the early ring stage. Pretreatment (1 h) with the haem synthesis precursor ALA (1 mM) enhanced the labelling signal intensity. Conversely, pretreatment with the haem synthesis inhibitor SA (500 μM) partially reduced the labelling intensity. (**c**) A model for artemisinin's mechanism of action. Artemisinin activation relies on haem generated in the parasite from both biosynthesis and haemoglobin digestion. In the early ring stage, biosynthesized haem was primarily responsible for drug activation; at the latter stages, both pathways co-existed, with haem derived from haemoglobin digestion playing the major role. COPROGEN, coproporphyrinogen III; CRT, chloroquine resistance transporter; ER, endoplasmic reticulum; Hb, haemoglobin; MDR, multidrug resistance protein; PBG, porphobilinogen; PPIX, protoporphyrin IX; PROTOGEN, protoporphyrinogen IX; UROGEN, uroporphophyrinogen III. Full-gel images for panels **a** and **b** are shown in Supplementary Fig. 13.

We next studied artemisinin activation at different asexual intraerythrocytic stages of the parasite. In agreement with the earlier finding that artemisinin is effective against the parasite at different stages^37^, we found that **AP1** targeted parasite proteins at all stages, but the effects were smaller against the early ring stage than the latter stages (trophozoite and schizont stages) (Fig. 5a). This is consistent with a previous report^38^ showing that the killing effect of artemisinin is up to 100 times higher at the latter stages than the early ring stage. Unlike the activation of **AP1** at the latter stages, the haem source for **AP1** activation at the early ring stage was not from haemoglobin digestion^29^,^38^, as it has not yet occurred at this stage^39^. This was further confirmed by the observation that ALLN had no inhibitory effect on **AP1** activation and binding at the early ring stage (Fig. 5a).

Is the parasite's haem biosynthesis pathway^40^,^41^,^42^,^43^,^44^ involved in artemisinin activation at the early ring stage? We tested the effects of the haem precursor ALA and the haem synthesis inhibitor SA on artemisinin activation at the early ring stage. The modulation of haem levels through its biosynthetic pathway correlated well with the extent of **AP1** activation and binding (Fig. 5b) at the early ring stage, similar to what we observed in the *ex vivo* cancer model. In addition, the results of ring-stage parasite viability assays showed that ALA pretreatment increased, while SA pretreatment reduced, the parasite-killing effect (Supplementary Fig. 9), consistent with the aforementioned fluorescent-labelling results. Thus, these results reveal a previously unknown mechanism for artemisinin activation in the parasite at the early ring stage. However, when we modulated haem levels using ALA and SA in unsynchronized parasites, there was no significant alteration in the fluorescence-labelling intensity (Supplementary Fig. 10). Furthermore, the IC_50_ of artesunate co-treated with ALA or SA determined using a standard 48-h growth inhibition assay were also similar (Supplementary Table 4). Our results are consistent with a previous 5-aminolevulinic acid synthase and ferrochelatase knockout study in *P. falciparum*^45^, which showed that the modulation of haem biosynthesis does not affect artemisinin activity when the drug treatment spans the entire parasite life cycle in the blood. This also suggests that compared with the level of haem released from haemoglobin, the level of biosynthesized haem is much lower and only plays a minimal role in artemisinin activation compared with haemoglobin digestion at the latter stages. Therefore, we propose that there are two pathways to activate artemisinin in the malaria parasite (Fig. 5c). Activation of artemisinin at the early ring stage relies mainly on the parasite's haem biosynthesis; however, the level of activation is rather low due to the low level of haem production by the parasite at this stage. At the latter stages, both of these two pathways may activate artemisinin, but haem released from the digested haemoglobin plays a major role in drug activation. Higher drug activity corresponds to the extremely high haem level at the latter stages (Fig. 5c), which makes artemisinin exceptionally potent against the parasite. This unique mode of artemisinin activation also accounts for the selective toxicity of the drug towards the parasite, as the activated drug is largely confined within the parasites and infected RBCs.

## Discussion

Our results on the mechanisms of artemisinin activation at different parasite stages and the drug's promiscuous targeting are consistent with recent reports of artemisinin resistance^46^,^47^ due to the PfKelch13 mutation, which results in a prolonged ring stage with an enhanced stress response^23^,^48^,^49^. The level of drug activation is low at the ring stage. Parasites with PfKelch13 mutations are able to overcome protein damage due to the drug modifications by activating the stress response; thus, they are selected as they have a higher capability to survive the drug treatment at the ring stage. On the other hand, the much higher level of drug activation at the latter stages triggers extensive protein modifications that act like an exploding bomb, inhibiting multiple key biological processes and eventually resulting in parasite death. Thus, it is less likely that the parasite develops resistance at the latter stages.

Notably, artemisinin resistance due to the PfKelch13 mutation is different from typical drug resistance caused by mutations in drug targets. The multi-targeting nature of artemisinin makes it difficult for *P. falciparum* to acquire resistance due to the need for mutations of multiple targets. Thus, it is not surprising that previous studies could not find the association of PfATP6 and TCTP (known targets) mutations with the emerging drug resistance^50^. Rather, PfKelch13 mutations enhance the parasites' capability to cope with the stress induced by artemisinin multi-targeting at the ring stage, at which point drug activation and drug pressure are relatively low; thus, these mutations can be selected.

To cope with artemisinin resistance due to a prolonged ring stage, we can either extend the length of drug treatment, as suggested by Tilley and co-workers^51^, or increase the drug's activation level at the ring stage. As we have shown, pretreatment with ALA enhances artemisinin activation. ALA has been widely used in photodynamic therapy, such as the treatment of skin cancer, for many years^52^. Thus, co-treating patients with both ALA and artemisinin might improve the drug's efficacy towards ring-stage parasites.

Our results indicate that the clickable artemisinin probe is useful for the identification of protein targets of the drug and the visualization of drug activation levels both *in vitro* and *in vivo*. However, our experimental conditions are not useful for identifying non-covalent targets. Recently, a reversible artemisinin target, PfPI3K, was reported to be related to drug resistance in the ring stage^53^. The existence of non-covalent artemisinin targets, possibly with many others yet to be identified, further supports the notion that artemisinin acts via a promiscuous targeting mechanism. It will be interesting to develop new artemisinin probes that can be used to capture transient artemisinin–protein interactions. It will also be interesting to determine the roles of each covalent and non-covalent binding target in artemisinin-mediated parasite killing in future studies.

We have demonstrated here that artemisinin activation is mainly haem dependent, which we believe resolves previous arguments regarding the source of iron required for the drug activation. Artemisinin activation at the early ring stage seems to rely on the parasite's haem biosynthesis, whereas drug activation depends on haemoglobin digestion as the main haem source at the latter parasite stages, which determines the high specificity of the drug towards the parasite. Our findings contribute to a better understanding of the mechanism of action of artemisinin, which may facilitate the development of better strategies to fight malaria and its emerging drug resistance.

## Methods

### Synthesis of AP1

Reagents were obtained commercially and were used without further purification, except where indicated. Dichloromethane and 1,2-dichloroethane were freshly distilled from calcium hydride under a nitrogen atmosphere. Tetrahydrofuran was dried by distillation with sodium as a drying agent and benzophenone as an indicator. Silica gel (230–400 mesh) was used in flash column chromatography. ^1^H and ^13^C NMR spectra were recorded on a Bruker ARX 300. Chemical shifts (δ p.p.m.) were determined with tetramethylsilane as the internal reference. Mass spectra were recorded on a Finnigan LCQ mass spectrometer.

The synthetic scheme is shown in Supplementary Fig. 1. The preparation of intermediate **1** follows previously described methods^54^ with slight modifications. NaBH_4_ (1.2 g, 32.0 mmol) was added to a stirred solution of artemisinin (6.0 g, 21.2 mmol) in CH_3_OH (50 ml) at 0 °C. After the resulting mixture was stirred at the same temperature for 3 h, the mixture was neutralized with glacial acetic acid while maintaining the temperature at 0 °C. The mixture was then concentrated by evaporating most of the CH_3_OH, and the resulting residue was diluted with cold water and stirred for 15 min at room temperature. The precipitate was collected, washed with water and dried to give the title compound as a white solid (5.0 g, 85.0% yield). ^1^H NMR (300 MHz, CDCl_3_) δ 5.60–5.48 (m, 1H), 5.30–4.73 (m, 1H), 2.80–2.61 (m, 1H), 2.38–2.37 (m, 1H), 1.86–1.48 (m, 8H), 1.44–1.43 (m, 3H), 1.30–1.26 (m, 3H), 0.97–0.89 (m, 6H); liquid chromatography–mass spectrometry (LC–MS; APCI) calcd for [M+H–H_2_O]^+^: 267.15, found: 267.10.

The preparation of intermediate **2** followed previous descriptions^55^ with slight modifications. Benzoyl chloride (3.2 ml, 27.6 mmol) was added to a stirred solution of dihydroartemisinin 1 (5.0 g, 17.6 mmol) in anhydrous dichloromethane (60 ml) and anhydrous pyridine (9.0 ml) at 0 °C. After stirring at room temperature for 16 h, citric acid solution (7% in water, 50 ml) was added. The organic layer was separated, and the aqueous layer was further extracted with ethyl acetate (10 ml). The combined organic layer was washed with citric acid solution (7% in water, 50 ml), followed by saturated NaHCO_3_ aqueous solution (50 ml) and water (50 ml). The organic layer was then dried and concentrated, and the residue was purified by column chromatography (hexane:ethyl acetate=10:1) to yield the title compound as a white solid (6.0 g, 88.0% yield). ^1^H NMR (300 MHz, CDCl_3_) δ 8.13 (m, 2H), 7.57–7.45(m, 3H), 6.02 (m, 1H), 5.53 (s, 1H), 2.76 (m, 1H), 2.40 (m, 1H), 2.08–0.93 (m, 19H), 0.99–0.98 (m, 3H), 0.93–0.92 (m, 3H); LC–MS (APCI) calcd for [M+Na]^+^: 411.18, found: 411.20.

The preparation of intermediate **3** followed previously described methods^56^,^57^ with slight modifications. A solution of intermediate **2** (4.2 g, 11.0 mmol) in anhydrous 1,2-dichloroethane (50 ml) was added dropwise via cannula to a stirred mixture of allyltrimethylsilane (8.8 ml, 56 mmol), anhydrous ZnCl_2_ (1.8 g, 13.2 mmol) and 4A molecular sieve powder in anhydrous 1,2-dichloroethane (50 ml) at 0 °C. After stirring at the same temperature for 3 h, the reaction mixture was diluted with ethyl acetate (300 ml) and washed with citric acid solution (5% in water, 100 ml), saturated NaHCO_3_ aqueous solution (100 ml) and brine (100 ml). The organic layer was dried and concentrated. The residue was purified by column chromatography (hexane:ethyl acetate=10:1) to give the key intermediate as a white solid (2.5 g, 75.0%). Next, BH_3_SMe_2_ (borane dimethylsulfide; 2.0 M in diethyl ether, 2.0 ml, 4.0 mmol) was added to a stirring solution of this key intermediate (1.04 g, 3.36 mmol) in anhydrous tetrahydrofuran (20 ml) at 0 °C. The reaction mixture was allowed to warm up to room temperature and stirred for 24 h. A suspension of NaBO_3_.4H_2_O (2.50 g, 16.25 mmol) in water (25 ml) was then slowly added to the reaction mixture, and the resulting suspension was stirred for 24 h. Water (25 ml) was added, and the mixture was extracted with CH_2_Cl_2_ (25 ml). The organic extracts were dried over Na_2_SO_4_, filtered and concentrated under reduced pressure to give a colourless oil, which was then purified by column chromatography (hexane:ethyl acetate=4:1 to hexane:ethyl acetate=2:1) to obtain intermediate **3** (0.66 g, 62.0% yield) as a white solid. ^1^H NMR (300 MHz, CDCl_3_) δ 5.53 (s, 1H), 4.24–4.23 (m, 1H), 3.73–3.67 (m, 2H), 2.65–2.64 (m, 1H), 2.33–2.29 (m, 2H), 2.06–1.19 (17 m, 3H), 0.97–0.80 (6 m, 3H).

A solution of intermediate 3 (326 mg, 1.0 mmol), hex-5-ynoic acid (38 mg, 0.37 mmol), diphenylphosphoryl azide (275 mg, 1.0 mmol) and Et_3_N (101 mg, 1.0 mmol) in anhydrous CH_3_CN (50 ml) was stirred at 55 °C overnight under nitrogen. The reaction mixture was allowed to cool to room temperature, and the solvent was removed under reduced pressure. The resulting residue was taken up in ethyl acetate (100 ml) and washed with citric acid (5% in water, 50 ml × 2), water (50 ml), saturated NaHCO_3_ aqueous solution (50 ml) and brine (50 ml). The organic extracts were dried over Na_2_SO_4_, filtered and concentrated to give a colourless oil, which was then purified by flash chromatography (hexane:ethyl acetate=7:3) to obtain AP1 (90 mg, 58.0% yield) as a white solid. ^1^H NMR (300 MHz, CDCl_3_) δ 5.29 (s, 1H), 4.79 (m, 1H), 4.18–4.10 (m, 3H), 3.30–3.28 (m, 2H), 2.67–2.64 (m, 1H), 2.43–2.25 (m, 4H), 2.06–1.73 (m, 5H), 170–1.56 (m, 8H), 1.40–1.25 (m, 5H),0.97–0.95 (m, 3H), 0.87–0.85 (d, 3H) (see Supplementary Fig. 11); high-resolution mass spectrometry (ESI) calculated for [M+H]^+^: 436.2694, found: 436.2700 (see Supplementary Fig. 12).

### Reagents and equipment used in biological experiments

In addition to the aforementioned synthesized chemicals, other reagents used in our biological experiments are listed below. Artemisinin, artesunate, streptavidin beads, methanol, acetonitrile (ACN), formic acid (FA), urea, phosphoric acid, DMSO, Tris [(1-benzyl-1H-1,2,3-triazol-4-yl) methyl]amine (TBTA), Tris (2-carboxyethyl) phosphine (TCEP), CuSO_4_, ALLN, haemin, L-ascorbic acid (vitamin C), Na_2_SO_4_, DFO, DFP, FeSO_4_, (IAA, *N*-ethylmaleimide, ALA, SA, 4,5-dihydroxy-1,3-benzenedisulfonic acid disodium salt (Tiron), 6-hydroxy-2,5,7,8-tetramethylchroman-2-carboxylic acid (Trolox), 2,2,6,6-tetramethyl-1-piperidinyloxy (TEMPO), LDH and PyrK assay kits were purchased from Sigma-Aldrich (St Louis, MO, USA). Rhodamine–azide was obtained from Jinglan Co. (Guangzhou, China). Methyl methanethiosulfonate (MMTS) was purchased from Pierce (Rockford, IL, USA). RPMI 1640, Dulbecco's modified eagle medium and BSA were purchased from Invitrogen (Carlsbad, CA, USA). Sequencing grade trypsin was obtained from Promega (Madison, WI, USA). Protease and phosphatase inhibitor cocktails were purchased from Roche (Basel, Switzerland). All the parasite cultures were maintained in malaria culture media (MCM) consisting of RPMI 1640 (Invitrogen, USA) supplemented with 0.5% Albumax II (Gibco, Auckland, New Zealand), 0.25% gentamycin (Gibco, USA), 0.005% hypoxanthine (Sigma-Aldrich), 0.03% L-glutamate (Sigma-Aldrich) and 1.25% healthy human erythrocytes (Interstate Blood Bank, USA). Unless otherwise indicated, all other reagents used for the biochemical methods were purchased from Sigma-Aldrich. In-gel fluorescence scanning of SDS–PAGE gels was carried out with a Typhoon 9410 fluorescence scanner (GE Healthcare; Buckinghamshire, UK), and where applicable, the gel lanes were quantified using ImageQuant (GE Healthcare).

### Parasite culture and synchronization

*P. falciparum* laboratory strain 3D7 (MRA-102, MR4/ATCC (American Type Culture Collection), Manassas, VA, USA and Thailand-derived field isolate ARS 270 (a kind gift from François Nosten, Shoklo Malaria Research Unit (SMRU), Mae Sot, Thailand) were continuously cultured in the above-mentioned MCM culture medium. Culture flasks were gassed with 3% CO_2_, 4% O_2_ and 93% N_2_, and incubated at 37 °C. Synchronization of parasite cultures was performed by incubating the cultures in 5% (w/v) D-sorbitol (Merck, Germany) at 37 °C for 10 min, after which the cells were washed twice with culture medium before transferring to new flasks. Synchronization will lyse erythrocytes containing mature stages of parasites (trophozoites and schizonts) through hypotonic solution, leaving the ring stage-infected and -uninfected erythrocytes in the culture. Giemsa-stained thin blood smears were examined before each experiment to check for parasitaemia and parasite stages.

### Inhibitory concentration determination

Synchronized ring-stage cultures were diluted with fresh erythrocytes and MCM to 1% parasitaemia and 1.25% haematocrit. The cultures were then incubated with artemisinin, artesunate or **AP1** at different concentrations for 48 h in a 96-well plate. Subsequently, the cells were stained with 1 μg ml^−1^ of Hoechst 33342 (Invitrogen) for 20 min at 37 °C in the dark, and parasitaemia was determined with a CyAn flow cytometer (Beckman Coulter). Plotting of the sigmoidal dose–response curve was performed with GraphPad Prism 5 using a four-parameter logistic curve (variable slope). Three experiments were performed to obtain the mean values presented.

### Modified ring-stage viability assay

Tightly synchronized ring-stage (0–6 h) cultures were diluted with fresh erythrocytes and MCM to 1% parasitaemia and 1.25% haematocrit. The cultures were then incubated with PBS, SA (500 μM) or ALA (1 mM) for 1 h in a six-well plate at 37 °C. Subsequently, the cells were aliquoted into 96-well plates and then incubated with varying concentrations of artesunate (0–500 nM) for another 4 h at 37 °C. Finally, the drugs were washed off three times with fresh malaria culture medium and then returned to the 37 °C incubator for another 43 h. Cells were stained with 1 μg ml^−1^ of Hoechst 33342 (Invitrogen) for 20 min at 37 °C in the dark, and the parasitaemia was determined with a CyAn flow cytometer (Beckman Coulter). Plotting of the sigmoidal dose–response curve was performed with GraphPad Prism 5 using a four-parameter logistic curve (variable slope). Three experiments were performed to obtain the mean values presented.

### *In situ* fluorescence labelling of *P. falciparum*

*P. falciparum* parasites (unsynchronized) were diluted to ∼5% parasitemia and cultured with 1.25% haematocrit in a 12-well plate (1 ml parasite culture) in the gassed incubation chamber. Increasing concentrations (5–1,000 nM) of **AP1** dissolved in DMSO (final concentration of DMSO was 0.2%) were used to culture the parasites for 4 h at 37 °C. An equal volume of DMSO was used as the negative control. After probe incubation, the cultures were transferred to Eppendorf tubes and centrifuged at 600*g* for 5 min to remove the medium. Subsequently, 0.05% saponin in PBS was added, and the tubes were inverted several times before incubating on ice for 10 min to lyse RBCs. The cell lysates were then centrifuged at 10,000 r.p.m. (9,300*g*) for 10 min to remove RBC proteins. The resulting pellets were washed with PI buffer (1 × protease inhibitors in PBS) and centrifuged at 10,000 r.p.m. (9,300*g*) to collect the parasite fractions. To ensure removal of the RBC proteins, pellets were further washed twice with the same buffer. Parasite proteins were extracted by suspending the pellets in 0.16% SDS in PBS with 1 × protease inhibitors (EDTA free, Roche) before a brief sonication (31% amplification, 2 s on, 2 s off for 2 min on ice). Clear lysates were obtained via centrifugation (9,300*g*), and the protein concentrations were determined by DC protein assay (Bio-Rad). Equal amounts (50 μg) of different treatment samples were used for fluorescent tagging. For each click reaction, rhodamine–azide (10 μM), TBTA ligand (100 μM, 100 × stock in DMSO), TCEP (1 mM, 100 × fresh stock in water) and CuSO_4_ (1 mM, 100 × stock in water) were sequentially added to the lysates. Samples were incubated and shaken at room temperature for 3 h. Next, tagged proteins were precipitated with acetone and air dried. 1 × Laemmli buffer (35 μl) was added to dissolve the pellets, and 15 μl of each sample was separated by SDS–PAGE on 12.5% polyacrylamide gels or 4–20% gradient gels (Bio-Rad). Following one-dimensional gel separation, gels were scanned using a Typhoon 9410 laser scanner (GE Healthcare), and the images were analysed with ImageQuant software. The fluorescence contrast was normalized against the DMSO control to minimize the background.

For competition assays, the parasites were pretreated with excessive artesunate (25 ×) for 30 min then together with **AP1** (200 nM) for 4 h. Probe-labelled proteins were visualized by click conjugation to the rhodamine–azide followed by SDS–PAGE separation and fluorescence scanning.

For free-radical scavenger co-treatment, the parasites were co-treated with various scavengers together with **AP1** for 4 h. The concentrations of the free-radical scavengers used in this experiment were: Tiron (1 mM), Trolox (400 μM) and TEMPO (1 mM). Similarly to the aforementioned fluorescent labelling, drug- or chemical-treated parasites were isolated from RBC and lysed. The probe-labelled proteins were then subjected to rhodamine–azide conjugation, SDS–PAGE separation and fluorescence scanning.

For DFO, DFP and ALLN pretreatment, the parasites were pretreated with different concentrations of DFO or DFP (100 or 500 μM, 30 min) or ALLN (5, 10 or 15 μM, 1 h). **AP1** was then added to the culture to co-treat for another 4 h. Similar to the aforementioned fluorescent-labelling step, drug- or chemical-treated parasites were isolated from RBCs and lysed. Probe-labelled proteins were then subjected to rhodamine–azide conjugation, SDS–PAGE separation and fluorescence scanning.

For haem modulator treatment in early ring-stage parasites, highly synchronized early ring-stage parasites were pretreated with ALA (1 mM) or SA (0.5 mM) or with a combination of ALA (1 mM) and SA (0.5 mM) for 1 h. **AP1** was then added and incubated together with the haem modulators for another 4 h. Probe-labelled proteins were then subjected to rhodamine–azide conjugation, SDS–PAGE separation and fluorescence scanning.

### Parasite labelling for target identification

Five millilitres of parasite cultures (unsynchronized, 5% parasitaemia, 1.25% haematocrit) were incubated with **AP1** (500 nM) for 4 h at 37 °C. Parasite culture containing 0.2% DMSO was used as a negative control. Similar to the above procedure for fluorescence scanning, parasites were isolated and purified for subsequent click chemistry to conjugate proteins with the biotin tag. For each reaction, biotin–azide (10 μM), TCEP (1 mM, 100 × fresh stock in water), TBTA ligand (100 μM, 100 × stock in DMSO) and CuSO_4_ (1 mM, 100 × stock in water) were sequentially added to the cell lysates, and the lysates were incubated at room temperature for 4 h with shaking. Then, clicked proteins were subjected to precipitation with acetone and air dried. Subsequently, the pellet was dissolved in 5 ml of 0.1% SDS in PBS and incubated with 50 μl of streptavidin beads (Sigma-Aldrich) under gentle mixing for 2 h at room temperature.

### On-bead digestion by trypsin

Beads were washed thrice with 1% SDS, urea (6 M) and PBS before being re-suspended in 100 μl triethylammonium bicarbonate (25 mM), in which 2 μl TCEP (100 mM stock solution) was then added. The beads were heated at 65 °C for 1 h before allowing the samples to react for 15 min at room temperature, after which 1 μl MMTS (200 mM stock solution) was added. With reduction and alkylation performed, trypsin (12.5 ng μl^−1^) was added to the samples, which were then incubated at 37 °C overnight. The beads were separated from the digested peptides with a filter-spin column (GE Healthcare).

### Liquid chromatography–mass spectrometry/mass spectrometry

The LC–MS/MS method was described previously^58^. Separation of the peptides was performed using an Eksigent nanoLC Ultra and ChiPLC-nanoflex (Eksigent, Dublin, CA, USA) in Trap-Elute configuration. Desalting of the samples was carried out with a Sep-Pak tC 18 μl Elution Plate (Waters, Milford, MA, USA) before reconstituting in 50 μl of diluent (98% water, 2% ACN and 0.1% FA). With the use of a 200 μm × 0.5 mm trap column, 5 μl of each sample was loaded and eluted on an analytical column (75 μm × 150 mm). Both trap and analytical columns were made of ChromXP C18-CL, 3 μm (Eksigent, Germany). At a flow rate of 300 nl min^−1^, the peptides were segregated by a gradient formed with mobile phase A (2% ACN, 0.1% FA) and mobile phase B (98% ACN, 0.1% FA): 5–12% of mobile phase B (20 min), 12–30% of mobile phase B (90 min), 30–90% of mobile phase B (2 min), 90% of mobile phase B (5 min), 90–5% of mobile phase B (3 min) and 5–5% of mobile phase B (13 min).

MS analysis was carried out with a TripleTOF 5600 system (SCIEX, Foster City, CA, USA) in information-dependent mode. A high-resolution mode (>30,000), which consists of 250-ms accumulation time per spectrum and a mass range of 400–1,250 *m*/*z*, was set before MS spectra were obtained. For each duty cycle/MS spectrum, a maximum of 20 precursors with a charge state between 2 and 4 was selected for fragmentation, with a 100-ms minimum accumulation time for each precursor and dynamic exclusion for 15 s.

The protein and peptide summary of artemisinin targets are shown in Supplementary Data 2–7.

The mass spectrometry proteomics data were deposited in the ProteomeXchange Consortium^59^ via the PRIDE partner repository with the data set identifier PXD002611.

### ProteinPilot analysis

The detailed methods of ProteinPilot analysis have been illustrated previously^60^. The proteins were identified with ProteinPilot 4.5 (SCIEX) that uses a Paragon algorithm to carry out database searches. PlasmoDB (v13, *P. falciparum* 3D7) was used as the database, while the search parameters used were as follows: cysteine alkylation with MMTS; trypsin digestion; TripleTOF 5600; and biological modifications. The identified proteins were grouped using the ProGroup algorithm in the software to remove any redundancy. To determine the false-discovery rate (FDR) for protein identification, a decoy database search strategy was utilized, of which a corresponding randomized database was produced using the Proteomics System Performance Evaluation Pipeline feature in the ProteinPilot 4.5 software. An unused score ≥1.3 was used as the cutoff threshold for protein identification, after which the FDR equals to 0.

### Mascot analysis

The mass spectrometric data were converted into Mascot generic format (MGF) using the ProteinPilot (SCIEX) software and then searched with Mascot 2.4.0 (Matrix Science). The database used was a combined database containing PlasmoDB (v13, *P. falciparum* 3D7) and human protein sequences (total of 95,492 sequences). The search parameters used were as follows: trypsin digestion; ESI-QUAD-TOF; acetyl (*N*-rterm), oxidation (M) and methylthio (C) as variable modifications; peptide mass tolerance was 100 p.p.m. and fragment mass tolerance was 0.4 Da; the maximum number of missed cleavages was 1. A decoy database search strategy was used to determine the FDR for peptide identification. We applied 1% FDR to filter the identified peptide list.

### Cloning, expression and purification of recombinant *P. falciparum* proteins

Purified genomic DNA from the *P. falciparum* 3D7 strain was obtained using an AxyPrep multisource genomic DNA miniprep kit (Corning, Tewksbury, MA, USA). The genes encoding *P. falciparum LDH*^61^, *OAT*^62^, *SAMS* and *TCTP*^63^, which do not have introns, were amplified by PCR directly using the genomic DNA as a template, while the genes encoding *PyrK* and *SpdSyn* were PCR amplified from the plasmids purchased from Addgene (PK_plasmid 25278 and SRM_plasmid 25110, Cambridge, MA, USA). The primers used for PCR amplification are listed in Supplementary Table 5. The PCR products were cloned into the pET28a vector (Novagen, Madison, WI, USA), and the encoded genes of interest in the constructs were confirmed by sequencing. To overexpress the recombinant proteins, the plasmids were then transformed into competent *E. coli* BL21 (DE3)-star cells (Invitrogen, Carlsbad, CA, USA). Ten millilitres of an overnight culture were diluted 1:100 in Luria–Bertani medium supplemented with 100 μg ml^−1^ ampicillin and grown at 37 °C until the OD_600_ reached ∼0.6. Expression of recombinant proteins was induced with 0.4 mM isopropyl β-D-1-thiogalactopyranoside. After overnight cultivation at 20 °C, the cells were collected by centrifugation and then re-suspended in B-per bacterial protein extraction reagent (Pierce, Thermo Scientific, Rockford, IL, USA) to extract the soluble proteins. After centrifugation, the supernatant was applied onto a Ni-NTA column (Thermo Scientific). The column was washed with buffer A (50 mM Tris-HCl, 300 mM NaCl, pH 7.4) followed by buffer B (buffer A with 10 mM imidazole, pH 7.4). The recombinant proteins were eluted with buffer C (buffer A with 250 mM imidazole, pH 7.4).

### Validation of drug targets using western blots

The **AP1** affinity pull-down sample was separated via one-dimensional SDS–PAGE together with the DMSO pull-down sample. After SDS–PAGE, the proteins were transferred onto polyvinylidene difluoride membranes (Bio-Rad). The blots were blocked with 5% (w/v) BSA in PBS with 0.1% Tween 20 (PBS-T) for 4 h at room temperature. The membranes were incubated with rabbit anti-actin (1: 500; Sigma), rabbit anti-MSP-1 (1:5,000), mouse anti-PM-I (1:5,000) and rabbit anti-PM-II (1:5,000). Horseradish peroxidase (HRP)-conjugated anti-rabbit (Pierce Biotechnology) or HRP-conjugated anti-mouse IgG (1:5,000; GE Healthcare) was used as a secondary antibody, and samples were incubated for 2 h at room temperature. The membrane was washed three times in PBS-T between each antibody incubation step. Subsequent visualization was performed using ECL substrate (Pierce Biotechnology).

### *In vitro* labelling of *P. falciparum* recombinant proteins

Recombinant OAT, TCTP, PyrK, SpdSyn, LDH and SAMS were reconstituted with PBS at 1 mg ml^−1^. Two microlitres of protein solution were used for labelling. To test the activation effects of **AP1** (20 μM) on the protein solution, a combination of different treatments involving haemin, L-ascorbic acid (Vc), Na_2_S_2_O_4_, FeSO_4_ and DFO (200 μM each) was used. The final reaction volume was topped up to 40 μl with PBS and incubated for 4 h at room temperature with shaking.

In this section, three main experiments were performed as follows. For concentration-dependent and time-dependent labelling, the proteins were treated with different doses of **AP1** and incubation durations in the presence of haemin and Vc. For the heat-denatured samples, the protein solution was dissolved in 1% SDS solution by diluting the sample with 1.2% SDS in PBS. After dissolving, the samples were heated at 96 °C for 10 min before cooling down to room temperature. **AP1** (20 μM), haemin and Vc were then added for labelling. A competition assay was carried out by pretreating the proteins with excess artesunate (25 ×) for 1 h in the presence of haem (haemin reduced by Vc) before incubating with **AP1** together with artesunate for another 4 h. To test whether the thiol and amine groups of the proteins react with the **AP1** probe, proteins were pretreated with 30 mM IAA or 10 mM *N*-ethylmaleimide for 20 min, and **AP1** was added to react for another 4 h.

All the aforementioned treatments and labelling were stopped by adding four volumes of pre-chilled acetone. The unreacted probe and other chemicals were removed after acetone precipitation of the proteins. The resulting pellets were dissolved in 0.16% SDS in PBS and tagged with the fluorescent dye for SDS–PAGE and fluorescence scanning.

### LDH inhibition assay

The assay followed the manufacturer's protocol with slight modifications. Purified LDH (1 μg) was first incubated with haemin (200 μM), Vc (200 μM) and different concentrations of artesunate (0–80 μM) in assay buffer (50 μl) at 37 °C for 4 h. The resulting mixture was transferred to a 96-well plate, followed by the addition of the reaction mix containing assay buffer (48 μl) and LDH substrate mix (2 μl). LDH activity was subsequently measured by absorbance at 450 nm with a microplate reader (Infinite M200 PRO, Tecan, Maennedorf, Switzerland). The reactions with no LDH added and with LDH without inhibitor served as negative and positive controls, respectively. All measurements were carried out in triplicate.

### Pyruvate kinase inhibition assay

The assays followed the manufacturer's protocol with slight modifications. Purified pyruvate kinase (1 μg) was first incubated with haemin (200 μM), Vc (200 μM) and different concentrations of artesunate (0–80 μM) in assay buffer (50 μl) at 37 °C for 4 h. The resulting mixture was transferred to a 96-well plate, followed by addition of the reaction mix containing assay buffer (44 μl), substrate mix (2 μl), enzyme mix (2 μl) and fluorescent peroxidase substrate (2 μl). Pyruvate kinase activity was subsequently measured by absorbance at 570 nm with a microplate reader (Infinite M200 PRO, Tecan). The reactions with no pyruvate kinase added and pyruvate kinase without inhibitor served as negative and positive controls, respectively. All measurements were carried out in triplicate.

### Pathway analysis

The GO analysis was conducted using Cytoscape 3.2.1 software^64^,^65^ with the ClueGO^66^ plugin. The database (GO *Plasmodium falciparum*) used for the GO analysis was obtained from the GO Consortium^67^. The GO analysis was conducted using a two-sided hypergeometric test with Bonferroni correction. The GO term levels were from five to ten. The minimum number of genes to form a cluster was set at two, while the minimum percentage of genes covered by our data set against the database was set at 10%. The rest of the settings were left as defaults. The resultant Fig. 2 shows the major biological processes of the identified protein targets of artemisinin, while Supplementary Fig. 7 shows the cellular localization and Supplementary Fig. 8 shows the molecular functions.

### Cancer cell (HCT 116) culture

The HCT116 cell line was obtained from ATCC (Manassas, VA, USA) and cultured in Dulbecco's modified eagle medium (Sigma-Aldrich) supplemented with 10% fetal bovine serum (Invitrogen) and 1 × antibiotic/antimycotic (Invitrogen). Cells were cultured at 37 °C supplemented with 5% CO_2_.

### Haem modulator treatment and *in vitro* killing assay of HCT116 cells

Approximately 20,000 HCT116 cells were plated in triplicate in a 96-well plate and incubated for 24 h before being subjected to pretreatment with **AP1** and various haem modulators. A concentration of 35 μM **AP1** was used to treat the cells. For haem modulator pretreatment, 1 mM ALA, 0.5 mM SA, 100 μM FeSO_4_ and 100 μM DFO were added for 1 h before being incubated together with artesunate for another 24 h. Cell viability was determined by the crystal violet assay as described previously^68^. Briefly, PBS was used to remove excess medium and dead cells that were still attached to the plate. The cells were then stained with 0.5% crystal violet in 20% methanol for 15 min. Excess crystal violet was washed off with PBS, and the plates were left to air dry. The cells were then solubilized with 1% SDS for 30 min, and the absorbance was measured at 550 nm. All the results (the cell survival rates) were normalized against the controls. The killing effect was calculated as 100% survival rate minus the respective cell survival rate of each treatment. All measurements were carried out in triplicate.

### Fluorescence scanning of HCT116 cells treated with the AP1 and haem modulators

HCT116 cells were cultured in a 12-well plate until reaching 75% confluence. Thirty-five micromolar **AP1** was used to treat the cells. Similar concentrations of haem modulators as mentioned in the killing assay (the section above) were used for the pretreatment (1 h) and incubated together with **AP1** for another 6 h. The cells were washed three times with PBS to remove any free probe and other compounds present. Cells were then lysed by lysis buffer (0.16% SDS in PBS) before the protein concentrations were determined with a DC protein assay (Bio-Rad). Approximately 20 μg proteins for each treatment were clicked with rhodamine–azide for fluorescence scanning. The florescence intensity of the gel was quantified using ImageQuant. All measurements were carried out in triplicate.

## Additional information

**Accession codes:** The mass spectrometry proteomics data have been deposited in the ProteomeXchange Consortium via the PRIDE partner repository with the data set identifier PXD002611.

**How to cite this article:** Wang, J. *et al.* Haem-activated promiscuous targeting of artemisinin in *Plasmodium falciparum*. *Nat. Commun.* 6:10111 doi: 10.1038/ncomms10111 (2015).

## Supplementary Material

Supplementary Information Supplementary Figures 1-13, Supplementary Tables 1-5 and Supplementary References

Supplementary Data 1 Full list of artemisinin targets and relative abundance information.

Supplementary Data 2 Peptide summary of artemisinin targets (replicate 1).

Supplementary Data 3 Protein summary of artemisinin targets (replicate 1).

Supplementary Data 4 Peptide summary of artemisinin targets (replicate 2).

Supplementary Data 5 Protein summary of artemisinin targets (replicate 2).

Supplementary Data 6 Peptide summary of artemisinin targets (replicate 3).

Supplementary Data 7 Protein summary of artemisinin targets (replicate 3).

## Figures and Tables

**Table 1:** Validated artemisinin targets and their functions.

**Target**	**Molecular functions and biological processes involved**
OAT	Ornithine metabolism, arginine and proline metabolism
PyrK	Glycolysis, pyruvate kinase activity
LDH	L-lactate dehydrogenase activity, cysteine and methionine metabolism
SpdSyn	Spermidine biosynthesis
SAMS	*S*-adenosylmethionine biosynthesis, methionine adenosyltransferase activity
TCTP*	Calcium binding and microtubule stabilization†

^*^TCTP is a known artemisinin target^12^. It was not within the list of our identified artemisinin targets, but it was validated using an *in vitro* pure protein-binding assay.

^†^The annotated function refers to human TCTP. The function of PfTCTP has not been well characterized.
