# Supplementary material for: Haem-activated promiscuous targeting of artemisinin in Plasmodium falciparum
Source: Nat Commun. 2015 Dec 22;6:10111. doi: 10.1038/ncomms10111 (PMC4703832; doi:10.1038/ncomms10111)
Supplement: Supplementary Information — Supplementary Figures 1-13, Supplementary Tables 1-5 and Supplementary References [file ncomms10111-s1.pdf]

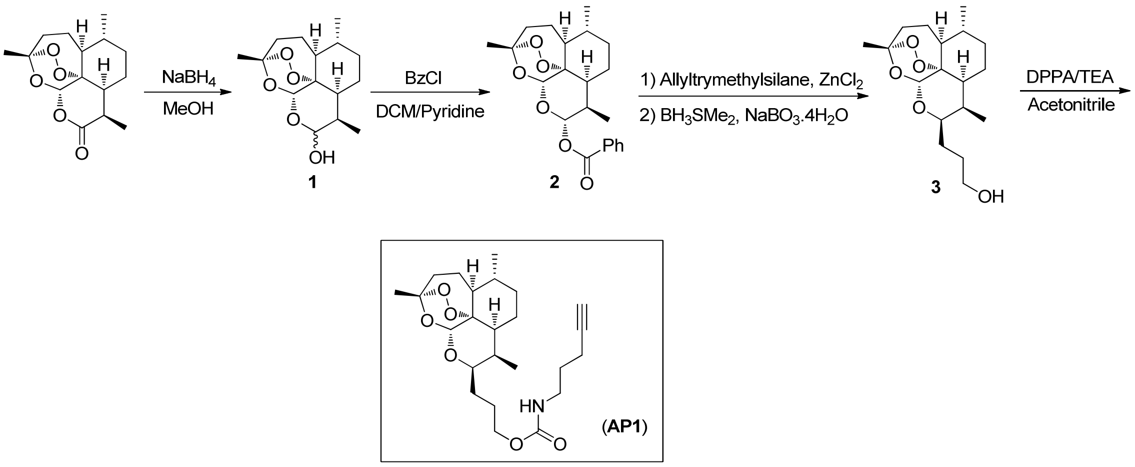

Supplementary Figure 1 | Synthetic scheme for the artemisinin-based probe AP1. Starting from the native artemisinin, **AP1** was synthesized through 5 steps with an overall yield of 26.8%.

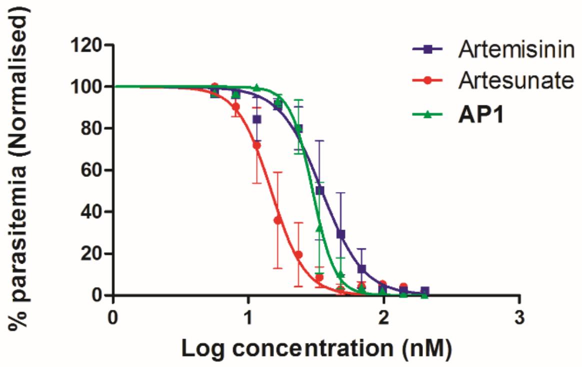

Supplementary Figure 2 | Dose-dependent parasite killing effects of AP1, artemisinin and artesunate on the *P. falciparum* field isolate ARS270. The killing effect of **AP1** is comparable with artemisinin and artesunate. Error bars represent s.d. in three independent replicates.

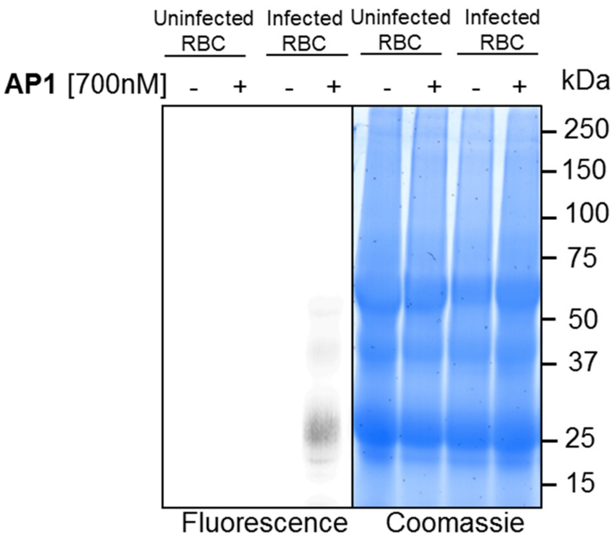

Supplementary Figure 3 | AP1 fluorescence labelling efficiency on uninfected and infected RBC proteins. AP1 does not label proteins of uninfected RBCs but can label some proteins of the infected RBCs. This may be due to the release of parasite-activated drugs into the cytosol of infected RBCs.

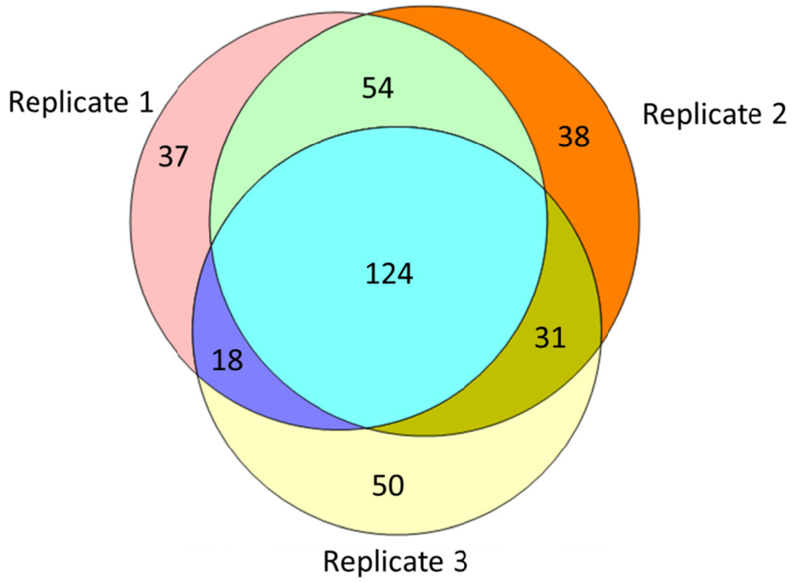

Supplementary Figure 4 | Venn diagram showing the numbers of protein targets identified from three independent pull-down experiments. The 124 proteins consistently identified in all three experiments were regarded as direct targets of artemisinin.

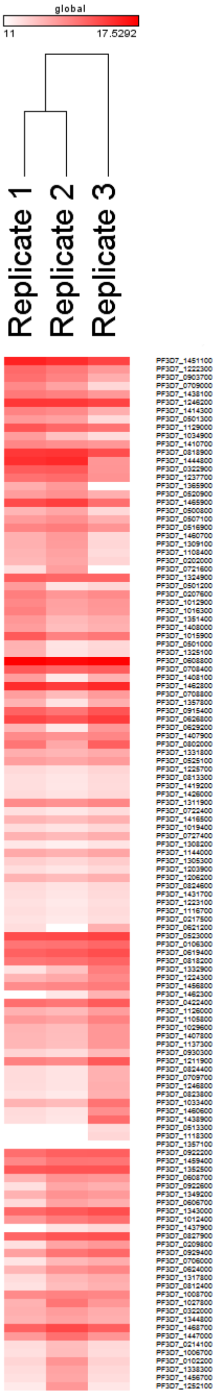

Supplementary Figure 5 | Heat map representation of the relative abundance of individual artemisinin target proteins. First, emPAI scores of the commonly identified 124 proteins from each individual replicate were used to calculate the respective Σ (emPAI × M_r). Subsequently, global normalization was performed on each individual (emPAI × M_r) value using the largest Σ(emPAI × M_r) as the common denominator. Finally, the Log_2(emPAI × M_r) values of individual proteins were used to generate the heat map with GENE-E software (www.broadinstitute.org/cancer/software/GENE-E/index.html), and the three groups of replicates were clustered by hierarchical clustering.

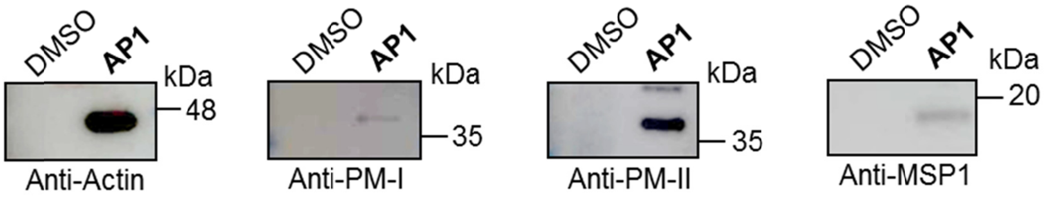

Supplementary Figure 6 | Western blot validation of pull-down fractions of parasite proteins by AP1 (or DMSO as negative control). Unsynchronized live parasites were treated with **AP1** followed by affinity pull-down. After SDS-PAGE separation, antibodies against selected targets were used to probe the respective proteins in the pull-down fraction. The results confirmed the specific pull-down of these protein targets by **AP1**. The full blot images are shown in Supplementary Figure 13.

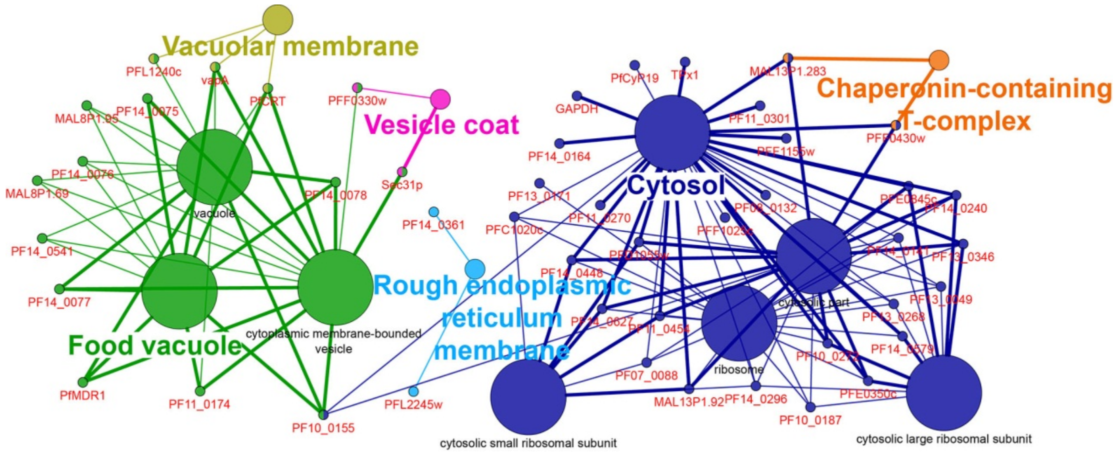

Supplementary Figure 7 | GO analysis for cellular localization of artemisinin targets using Cytoscape with the Cluego plugin. The results show that proteins targeted by artemisinin are not localized to a specific cellular region; rather, they are found in cellular locations, such are the vacuolar membrane, food vacuole, cytosol, and rough endoplasmic reticulum membrane, as shown in the figure. This shows that artemisinin is able to translocate to different regions of the malaria parasite to bind to its protein targets, highlighting its potential for differential effects on the physiological state of the malaria parasite.

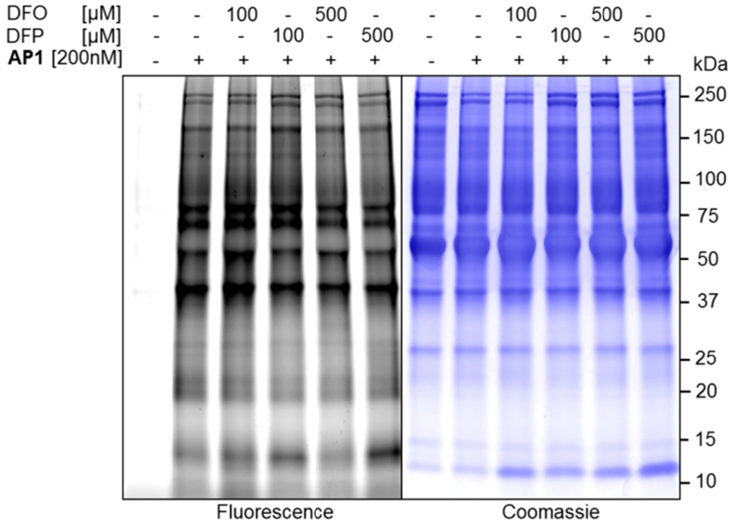

Supplementary Figure 8 | Effects of the iron chelators DFO and DFP on artemisinin activation. Unsynchronized live parasites were treated with **AP1** in the absence or presence of DFO or DFP and visualized with fluorescence labelling by SDS-PAGE. Both DFO and DFP do not have significant effects on artemisinin activation, as evidenced by similar fluorescence labelling intensities of **AP1** with or without DFO or DFP treatment.

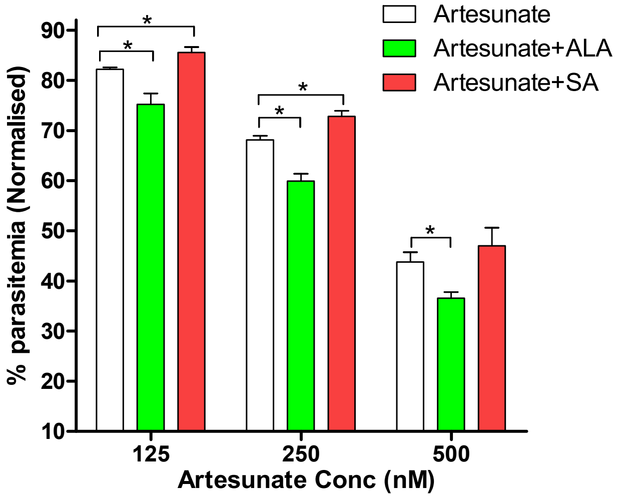

Supplementary Figure 9 | Dose-dependent parasite killing effect of artesunate in the early ring stage in the presence of haem synthesis modulators. The haem synthesis precursor ALA significantly enhances the parasite killing effect of artesunate, while the haem synthesis inhibitor SA attenuates the killing effect. * indicates $p < 0.05$. Error bars represent s.d. in three independent replicates.

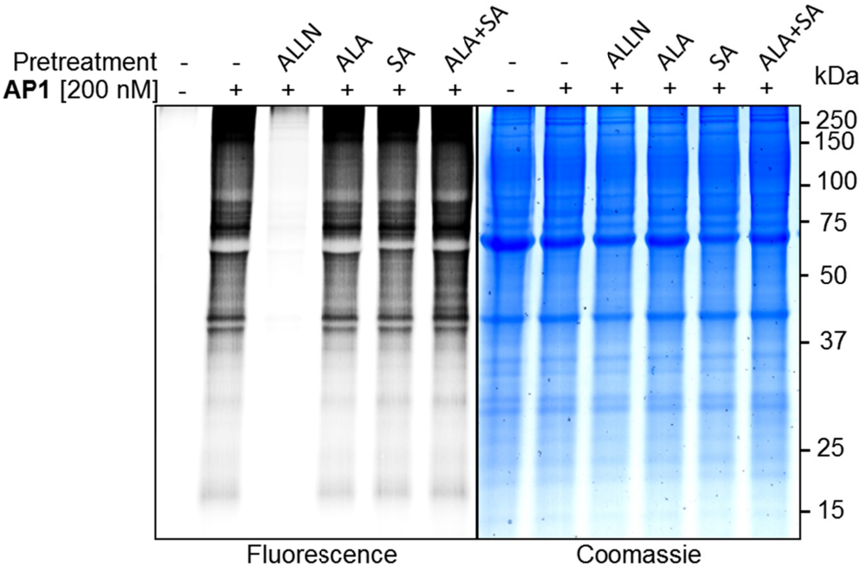

Supplementary Figure 10 | Effects of various haem modulators on the fluorescence labelling profile of AP1 in unsynchronized parasites. Both the haem synthesis precursor ALA and haem synthesis inhibitor SA, separately or in combination, did not have any significant effect on the fluorescence labelling intensity, suggesting that modulating haem biosynthesis does not affect drug activation in unsynchronized parasites. Conversely, the cysteine protease inhibitor ALLN dramatically attenuated the fluorescence signals, indicating that haem derived from haemoglobin digestion plays a major role in artemisinin activation in unsynchronized parasites.

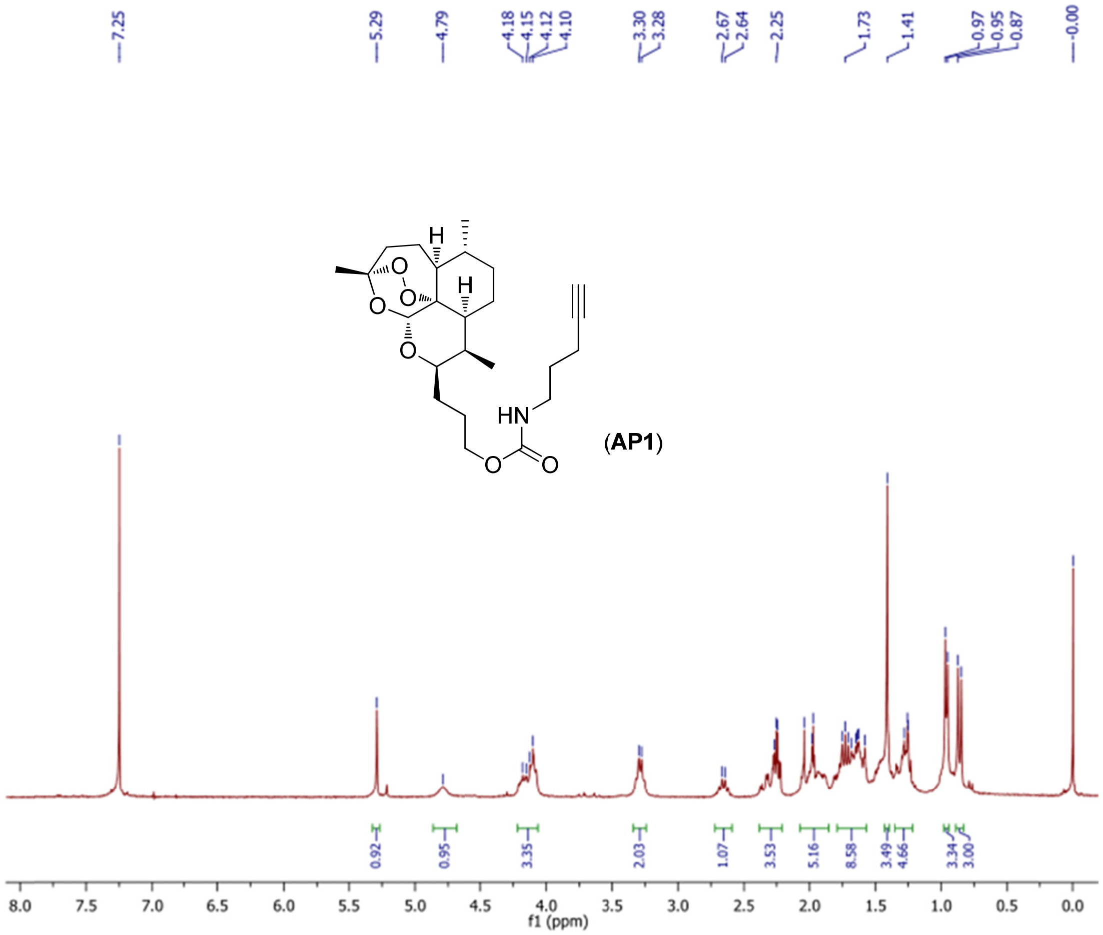

Supplementary Figure 11 | ^{1}H NMR spectrum of the artemisinin-based probe AP1. The probe was dissolved in $CDCl_3$ and the spectrum was recorded on a Bruker ARX 300.

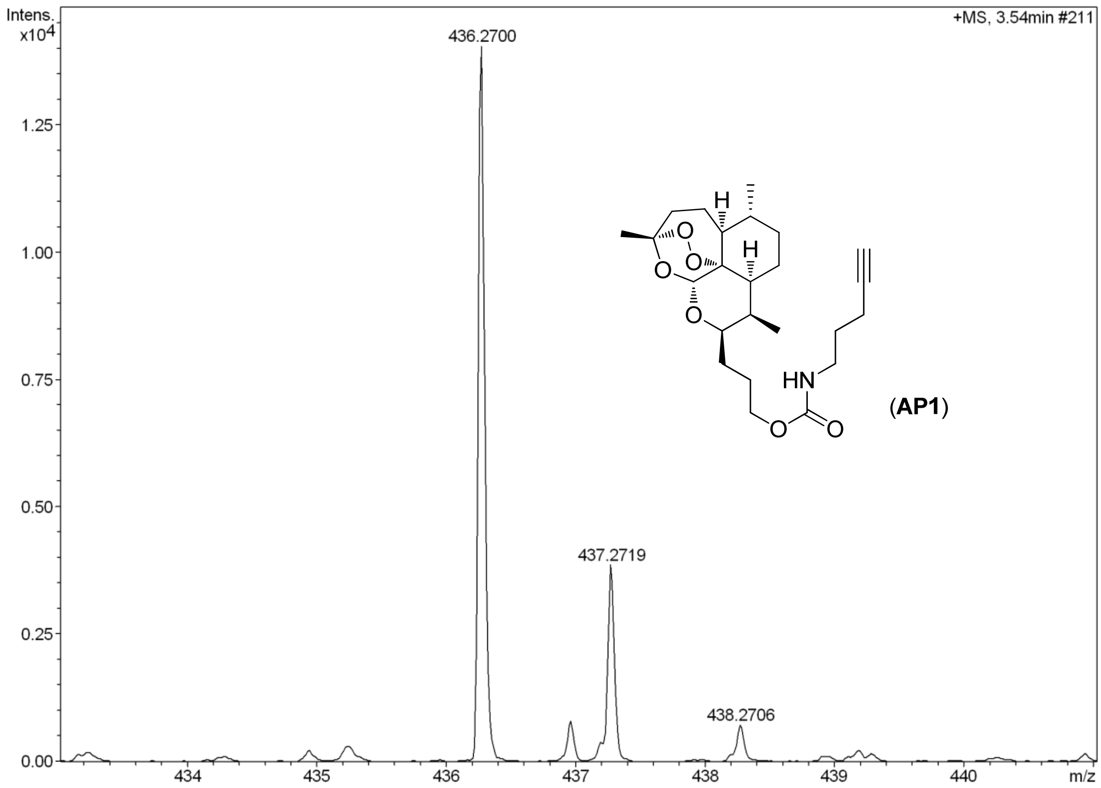

Supplementary Figure 12 | High resolution mass spectrum of the artemisinin-based probe AP1. ESI mass spectrum was recorded with an AmaZon X LC-MS.

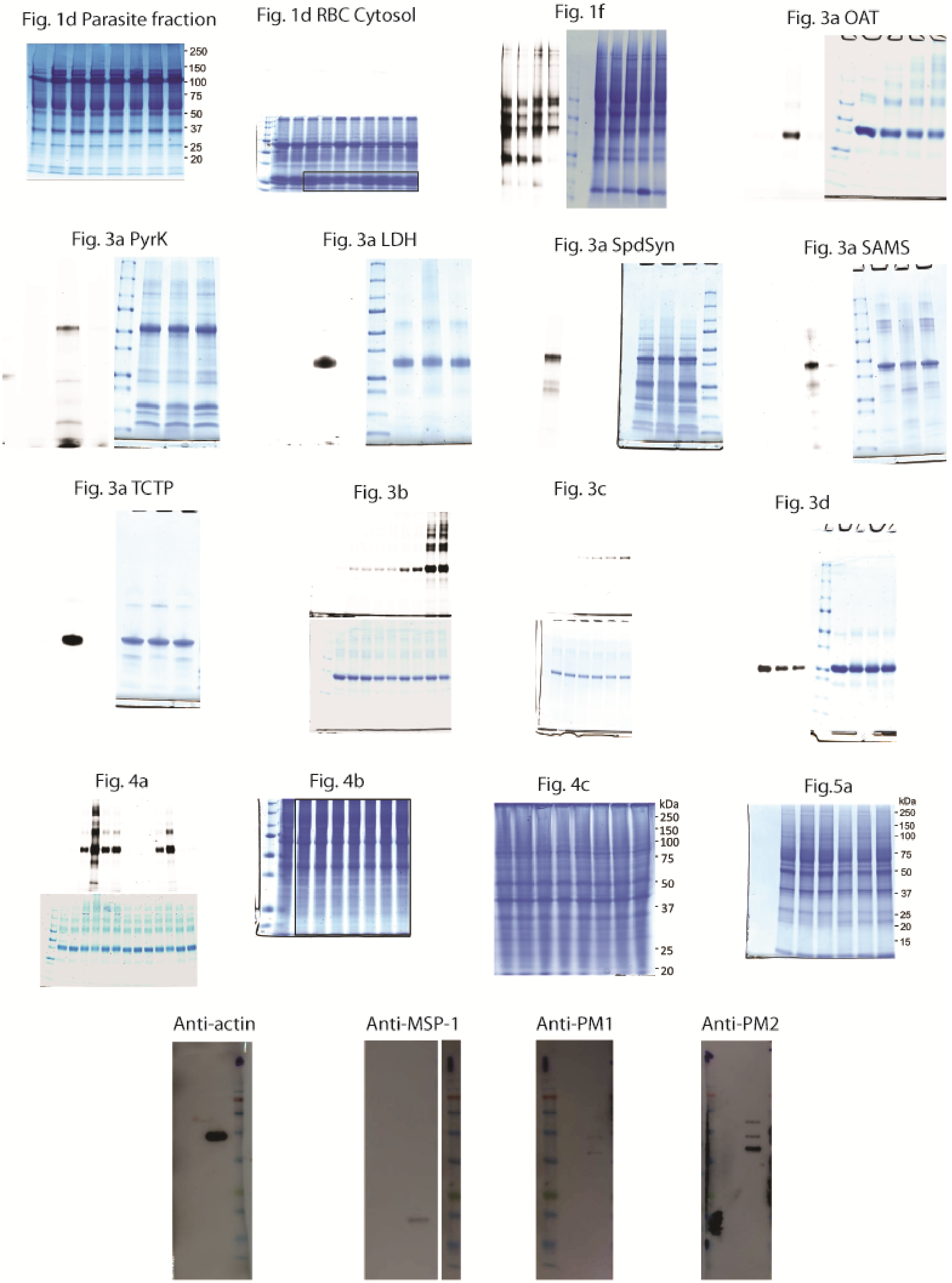

Supplementary Figure 13 | Full size gels and western blots. Only cropped blots and gels are shown.

Supplementary Table 1 | The artemisinin targets identified by AP1. All the targets have been identified from three separate pull-down LC-MS/MS experiments. No *P. falciparum* proteins were identified from the DMSO-treated control pull-down samples (a detailed and searchable target list is included in Supplementary Data 1).

No.	Accession No.	Protein Name	Replicate 1			Replicate 2			Replicate 3		
			score[1]	%Cov[2]	Pep.[3]	score[1]	%Cov[2]	Pep.[3]	score[1]	%Cov[2]	Pep.[3]
1	PF3D7_0608800	ornithine aminotransferase (OAT)	57.8	78.3	40	51.2	83.3	36	53.6	69.3	41
2	PF3D7_1357100	elongation factor 1-alpha	34.5	52.1	20	36.1	58.7	23	33.9	49	22
3	PF3D7_0523000	multidrug resistance protein (MDR1)	33.8	20.2	18	29.4	23.8	18	26.6	24.5	14
4	PF3D7_1451100	elongation factor 2	30.7	26.7	16	33.3	31	19	32.4	34.3	18
5	PF3D7_0818900	heat shock protein 70 (HSP70)	29.2	33.1	15	32.8	31.8	17	22.4	35.5	13
6	PF3D7_1246200	actin I (ACT1)	28.4	48.7	19	26.9	58.5	19	18.7	54.8	20
7	PF3D7_1462800	glyceraldehyde-3-phosphate dehydrogenase (GAPDH)	27.1	49	14	22.0	46.9	12	31.7	54.6	19
8	PF3D7_1444800	fructose-bisphosphate aldolase (FBPA)	25.3	41.7	13	26.2	45.8	15	7.2	18.7	4
9	PF3D7_0619400	cell division cycle protein 48 homologue, putative	24.5	26	12	23.6	31.4	12	14.1	24	9
10	PF3D7_0708400	heat shock protein 90 (HSP90)	21.5	19.6	11	17.2	21.6	9	12.4	18.7	7
11	PF3D7_1468700	eukaryotic initiation factor 4A (eIF4A)	21.3	36.9	14	31.2	49	21	24.7	48.5	17
12	PF3D7_0626800	pyruvate kinase (PyrK)	21.2	28.8	13	26.5	38.8	14	25.3	48.1	14
13	PF3D7_1324900	L-lactate dehydrogenase (LDH)	20.9	46.8	10	18.0	41.8	10	10.0	41.5	5
14	PF3D7_0827900	protein disulfide isomerase (PDI8)	19.0	37.3	9	17.7	37.3	11	22.3	40.6	11
15	PF3D7_1352500	thioredoxin-related protein, putative	18.9	31.7	16	16.8	40.9	16	19.7	37.5	19
16	PF3D7_1129000	spermidine synthase (SpdSyn)	18.3	26.2	10	13.9	32.7	7	10.9	18.4	6
17	PF3D7_0915400	6-phosphofructokinase (PFK9)	18.0	12.9	9	17.7	15.5	10	20.5	14.7	10
18	PF3D7_0802000	glutamate dehydrogenase, putative (GDH3)	16.3	12.6	8	7.5	8.8	5	18.8	18.2	10
19	PF3D7_1222300	endoplasmin, putative (GRP94)	15.3	17.8	8	12.4	15.4	6	7.1	19.5	4
20	PF3D7_1012400	hypoxanthine-guanine phosphoribosyltransferase	15.2	55	7	15.3	59.7	10	14.0	48.5	7

		(HGPRT)									
21	PF3D7_1015900	enolase (ENO)	14.4	32.3	8	8.5	24	4	15.9	28	8
22	PF3D7_1465900	40S ribosomal protein S3	14.3	38.5	7	19.8	51.1	10	8.1	30.3	4
23	PF3D7_1211900	non-SERCA-type Ca2+ -transporting P-ATPase (ATP4)	13.5	11.4	7	11.4	13.8	7	18.2	18.7	9
24	PF3D7_0903700	alpha tubulin 1	13.0	24.7	6	10.0	20.1	5	3.9	14.4	2
25	PF3D7_0202000	knob-associated histidine-rich protein (KAHRP)	12.7	17.3	12	15.7	21.6	13	6.0	12.8	4
26	PF3D7_0922200	S-adenosylmethionine synthetase (SAMS)	12.2	20.9	6	12.7	34.1	6	12.2	32.6	7
27	PF3D7_1456800	V-type H(+)-translocating pyrophosphatase, putative (VP1)	12.2	13.5	6	14.2	17.6	8	9.2	13.1	5
28	PF3D7_0507100	60S ribosomal protein L4 (RPL4)	12.1	24.6	6	10.0	15.3	5	8.1	20.7	4
29	PF3D7_1343000	phosphoethanolamine N-methyltransferase (PMT)	12.0	27.4	6	16.0	51.1	8	14.0	36.5	7
30	PF3D7_0322900	40S ribosomal protein S3A, putative	11.4	25.6	8	12.4	35.5	7	3.7	27.9	2
31	PF3D7_1237700	conserved Plasmodium membrane protein, unknown function	11.2	29.1	7	13.3	29.5	9	7.4	16.7	5
32	PF3D7_1027800	60S ribosomal protein L3 (RPL3)	10.5	24.6	6	8.7	28	6	8.5	24.1	5
33	PF3D7_1459400	conserved Plasmodium protein, unknown function	10.4	26.2	5	11.2	34.1	7	12.0	32.4	6
34	PF3D7_0929400	high molecular weight rhoptry protein 2 (RhopH2)	10.3	7	5	19.7	13.9	10	13.1	14.8	6
35	PF3D7_0207600	serine repeat antigen 5 (SERA5)	10.1	12.1	6	7.5	8.7	3	9.0	8.7	5
36	PF3D7_0422400	40S ribosomal protein S19 (RPS19)	10.0	36.5	5	12.5	50	7	10.5	44.1	5
37	PF3D7_1016300	glycophorin binding protein (GBP)	9.5	59.1	5	6.0	47.5	3	4.6	48.2	2
38	PF3D7_0106300	calcium-transporting ATPase (ATP6)	9.2	14.4	6	12.5	14.3	7	10.3	13.4	5
39	PF3D7_1311900	vacuolar ATP synthase subunit a (vapA)	8.3	11.8	4	8.0	13.9	4	7.1	13.1	4
40	PF3D7_0516900	60S ribosomal protein L2 (RPL2)	8.0	33.9	4	8.0	34.6	4	4.0	16.5	2
41	PF3D7_0525100	acyl-CoA synthetase (ACS10)	8.0	9.7	4	7.9	9.1	4	5.0	15	3
42	PF3D7_1407800	plasmepsin IV (PM4)	8.0	16	4	5.4	11.1	3	10.3	19.6	6
43	PF3D7_1407900	plasmepsin I (PMI)	8.0	25.9	4	6.8	17.7	4	5.2	15.9	5
44	PF3D7_1410700	conserved Plasmodium protein, unknown function	8.0	18.6	4	6.3	20.1	5	5.3	11.1	3
45	PF3D7_0709000	chloroquine resistance transporter (CRT)	7.6	12.3	5	7.6	15.1	4	2.9	11.3	3

46	PF3D7_1105800	conserved Plasmodium protein, unknown function	7.6	22.9	5	6.5	29.7	4	4.3	26.3	2
47	PF3D7_0818200	14-3-3 protein (14-3-3I)	7.0	30.2	5	10.0	39.3	7	9.0	29.4	8
48	PF3D7_1332900	isoleucine--tRNA ligase, putative	6.9	4.7	3	3.4	5.6	1	9.8	11.9	6
49	PF3D7_0501000	Plasmodium exported protein, unknown function	6.7	15.4	4	3.2	11.2	3	2.0	7.7	1
50	PF3D7_1008700	tubulin beta chain	6.2	15.5	4	10.0	18.9	5	8.0	20.9	4
51	PF3D7_1460700	60S ribosomal protein L27 (RPL27)	6.2	21.9	3	8.0	21.9	4	2.0	6.8	1
52	PF3D7_1438100	secretory complex protein 62 (SEC62)	6.2	17.2	4	8.1	19.6	4	5.2	23.9	3
53	PF3D7_1012900	autophagy-related protein 18, putative (ATG18)	6.2	18.7	3	7.7	15.5	4	8.1	21.6	4
54	PF3D7_0500800	mature parasite-infected erythrocyte surface antigen,erythrocyte membrane protein 2 (MESA)	6.1	10	3	12.0	15.6	6	2.1	10.7	1
55	PF3D7_0706000	importin-7, putative	6.0	4.7	3	4.0	5.8	2	2.2	8.8	1
56	PF3D7_0812400	karyopherin alpha (KARalpha)	6.0	8.6	3	9.6	18	5	5.3	10.8	3
57	PF3D7_0322000	peptidyl-prolyl cis-trans isomerase (CYP19A)	6.0	22.8	4	6.0	40.9	4	4.0	35.1	2
58	PF3D7_0629200	DnaJ protein, putative	6.0	10.8	3	2.3	10.3	1	3.5	16.8	2
59	PF3D7_0930300	merozoite surface protein 1 (MSP1)	6.0	3.5	3	2.0	3.5	1	2.0	3.5	1
60	PF3D7_1349200	glutamate--tRNA ligase, putative	6.0	7.5	3	8.8	10.3	4	8.2	11.9	5
61	PF3D7_1357800	TCP-1/cpn60 chaperonin family, putative	6.0	12.9	3	2.8	12.3	2	6.3	20	3
62	PF3D7_1408000	plasmepsin II	6.0	14.6	3	4.0	17.9	2	5.2	19.4	3
63	PF3D7_0608700	chaperone, putative	5.7	5.9	3	4.8	10.5	4	5.0	12.7	3
64	PF3D7_1305300	conserved Plasmodium protein, unknown function	5.6	2.8	3	2.0	5.1	1	2.0	5	1
65	PF3D7_1252100	rhoptry neck protein 3 (RON3)	5.5	6	3	10.2	5.9	5	2.0	4.7	1
66	PF3D7_1447000	40S ribosomal protein S5	5.5	37.9	2	12.3	39	7	8.0	28.3	5
67	PF3D7_0501300	skeleton-binding protein 1 (SBP1)	5.4	17.2	3	6.3	24.3	4	1.6	10.1	1
68	PF3D7_0513300	purine nucleoside phosphorylase (PNP)	4.6	14.3	2	2.5	9	1	2.0	9.8	1
69	PF3D7_0520900	S-adenosyl-L-homocysteine hydrolase (SAHH)	4.5	10.2	2	9.9	19.4	5	4.0	10.4	2
70	PF3D7_1029600	adenosine deaminase (ADA)	4.5	13.6	2	4.7	15.8	2	6.0	18	3
71	PF3D7_1034900	methionine--tRNA ligase, putative	4.3	12.3	2	4.3	13.7	2	4.1	11.6	2
72	PF3D7_0823800	DnaJ protein, putative	4.2	7.3	2	3.8	10.7	2	2.5	10.5	1

73	PF3D7_1462300	conserved Plasmodium protein, unknown function	4.2	4.5	2	1.8	5.4	2	3.0	11	2
74	PF3D7_1308200	carbamoyl phosphate synthetase (cpsSII)	4.1	3.5	2	3.5	4.1	2	1.4	4.6	1
75	PF3D7_1344800	aspartate carbamoyltransferase (ATCase)	4.1	11.2	2	4.1	13.1	2	3.9	14.1	2
76	PF3D7_1246800	signal recognition particle receptor, beta subunit (SRPR-beta)	4.1	12.3	2	2.8	15.3	2	4.1	13.8	2
77	PF3D7_0501200	parasite-infected erythrocyte surface protein (PIESP2)	4.0	11.5	2	2.0	15.9	1	2.0	3.7	1
78	PF3D7_0606700	coatomer alpha subunit, putative	4.0	3.1	2	4.9	5.6	3	2.6	6.8	2
79	PF3D7_0624000	hexokinase (HK)	4.0	11.8	2	8.0	16.4	4	9.1	31.6	5
80	PF3D7_0727400	proteasome subunit alpha type-5, putative	4.0	33.6	2	4.0	23.8	2	4.5	28.1	3
81	PF3D7_1033400	haloacid dehalogenase-like hydrolase (HAD1)	4.0	12.9	2	4.0	9	2	7.5	21.5	4
82	PF3D7_1118300	insulinase, putative	4.0	10.6	2	2.5	6.6	1	4.0	9.6	2
83	PF3D7_1137300	conserved Plasmodium membrane protein, unknown function	4.0	8.4	2	4.0	7.5	2	5.8	9.3	3
84	PF3D7_1144000	40S ribosomal protein S21 (RPS21)	4.0	24.4	2	2.8	24.4	2	3.1	24.4	2
85	PF3D7_1203900	ubiquitin-conjugating enzyme E2 (UBC)	4.0	19.7	2	3.8	19.7	2	2.0	12.2	1
86	PF3D7_1206200	eukaryotic translation initiation factor 3 subunit 8, putative	4.0	8.7	2	1.4	6.6	1	2.1	10	2
87	PF3D7_1309100	60S ribosomal protein L24, putative	4.0	16.1	2	3.0	27.2	2	2.0	17.3	1
88	PF3D7_1331800	60S ribosomal protein L23, putative	4.0	16.6	2	4.0	23.7	2	3.2	16.6	2
89	PF3D7_1351400	60S ribosomal protein L17, putative	4.0	12.3	2	4.0	23.7	2	2.0	12.8	2
90	PF3D7_1456700	conserved Plasmodium protein, unknown function	4.0	3.2	2	3.2	11.4	1	2.0	2	1
91	PF3D7_1416500	NADP-specific glutamate dehydrogenase (GDH1)	3.6	6.2	2	2.7	11.1	1	4.0	7	2
92	PF3D7_1408100	plasmepsin III,histo-aspartic protease (HAP)	3.5	9.5	2	2.0	4.9	1	4.0	5.5	2
93	PF3D7_0708800	heat shock protein 110 (HSP110c)	3.4	14	2	2.4	7.6	1	6.0	16	3
94	PF3D7_1414300	60S ribosomal protein L10, putative	3.2	19.2	2	2.9	20.6	2	4.0	8.7	2
95	PF3D7_0709700	lysophospholipase, putative	3.1	11.4	2	2.7	16.9	2	4.4	23.6	2
96	PF3D7_1224300	polyadenylate-binding protein, putative (PABP)	2.9	11.8	2	8.8	14.4	4	6.5	19.7	4
97	PF3D7_1365900	ubiquitin-60S ribosomal protein L40	2.4	31.3	1	4.3	31.3	2	2.0	21.1	2
98	PF3D7_1325100	phosphoribosylpyrophosphate synthetase	2.2	9.2	1	2.4	11	2	2.0	6.9	1

99	PF3D7_1225700	conserved Plasmodium protein, unknown function	2.1	6.3	1	2.0	5.2	1	1.9	2.3	1
100	PF3D7_1438900	thioredoxin peroxidase 1 (Trx-Px1)	2.1	18	1	2.0	17.4	1	6.2	42.6	5
101	PF3D7_0214100	protein transport protein SEC31 (SEC31)	2.1	4.4	1	4.1	3.9	2	2.0	4.8	1
102	PF3D7_1116700	cathepsin C, homolog,dipeptidyl aminopeptidase 1 (DPAP1)	2.0	4	1	2.7	9	2	1.3	14.7	2
103	PF3D7_0102200	ring-infected erythrocyte surface antigen (RESA)	2.0	3.2	1	7.4	8.9	4	3.9	7.2	2
104	PF3D7_0209800	ATP-dependent RNA helicase UAP56 (UAP56)	2.0	2.2	1	4.0	10.5	2	7.3	21.9	4
105	PF3D7_0217500	calcium-dependent protein kinase 1 (CDPK1)	2.0	8	1	3.5	18.7	2	2.6	8.6	2
106	PF3D7_0722400	GTP-binding protein, putative	2.0	3.6	1	1.9	5.9	1	1.9	11.2	1
107	PF3D7_0813300	conserved Plasmodium protein, unknown function	2.0	7.9	1	2.0	7.9	1	1.4	11.4	1
108	PF3D7_0824400	nucleoside transporter 2 (NT2)	2.0	2.2	1	2.0	6.2	1	4.0	6.2	2
109	PF3D7_0824600	Fe-S cluster assembly protein DRE2, putative (DRE2)	2.0	12.4	1	2.0	12.4	1	2.0	20.7	1
110	PF3D7_0922600	glutamine synthetase, putative	2.0	4.6	1	5.0	9.4	3	6.2	20.1	3
111	PF3D7_1006700	conserved Plasmodium protein, unknown function	2.0	4.4	1	6.0	9.4	3	2.0	6	1
112	PF3D7_1019400	60S ribosomal protein L30e, putative	2.0	12	1	2.0	12	1	2.0	26.9	1
113	PF3D7_1108400	casein kinase 2, alpha subunit (CK2alpha)	2.0	4.2	2	5.3	10.8	3	2.0	10.8	1
114	PF3D7_1126000	threonine--tRNA ligase (ThrRS)	2.0	4.3	1	2.0	4.3	1	4.3	10.5	2
115	PF3D7_1223100	cAMP-dependent protein kinase regulatory subunit (PKAr)	2.0	8.6	1	2.0	10.7	1	2.0	7.7	1
116	PF3D7_1317800	40S ribosomal protein S19 (RPS19)	2.0	7.6	1	2.6	24.8	1	4.0	22.8	2
117	PF3D7_1338300	elongation factor 1-gamma, putative	2.0	11.2	1	5.9	20.4	2	2.1	14.1	1
118	PF3D7_1419200	thioredoxin-like protein, putative	2.0	4.5	1	2.0	10	1	2.0	6.4	1
119	PF3D7_1426000	60S ribosomal protein L21 (RPL21)	2.0	14.9	1	2.0	8.1	1	2.0	14.9	1
120	PF3D7_1431700	60S ribosomal protein L14, putative	2.0	15.2	1	2.0	7.9	1	2.0	12.1	1
121	PF3D7_1460600	inner membrane complex sub-compartment protein 3, putative (ISP3)	2.0	10.1	1	2.0	10.1	1	4.4	28.4	3
122	PF3D7_0721600	40S ribosomal protein S5, putative	1.7	8.7	1	3.8	13.9	2	3.1	15.4	2
123	PF3D7_1437900	HSP40, subfamily A, putative	1.7	4.5	1	3.8	5.9	2	2.0	19.1	1

124	PF3D7_0621200	pyridoxine biosynthesis protein PDX1 (PDX1)	1.5	13.3	1	4.0	16	2	4.2	25.9	2

1. Score, total protein score. For the target identification, a strict total score cut-off of 1.3 was set as the qualification criterion, which corresponded to a protein confidence interval of 95%.

2. %Cov, percent protein sequence coverage with the identified peptides.

3. Pep., number of unique peptides identified for a protein.

Supplementary Table 2 | The artemisinin targets identified by AP1 that have been previously proposed as promising anti-malaria targets.

No.	Accession No.	Protein Name	References
1	PF3D7_0608800	ornithine aminotransferase (OAT)	[1,2]
2	PF3D7_0523000	multidrug resistance protein (MDR1)	[3]
3	PF3D7_1444800	fructose-bisphosphate aldolase	[4–7]
4	PF3D7_0626800	pyruvate kinase (PyrK)	[5]
5	PF3D7_1324900	L-lactate dehydrogenase (LDH)	[6]
6	PF3D7_1352500	thioredoxin-related protein, putative	[4]
7	PF3D7_1129000	spermidine synthase (SpdSyn)	[4,6]
8	PF3D7_0802000	glutamate dehydrogenase, putative (GDHc)	[2]
9	PF3D7_1012400	hypoxanthine-guanine phosphoribosyltransferase (HGPRT)	[4–7]
10	PF3D7_0922200	S-adenosylmethionine synthetase (SAMS)	[8]
11	PF3D7_0106300	calcium-transporting ATPase (ATP6)	[9,10]
12	PF3D7_0520900	S-adenosyl-L-homocysteine hydrolase (SAHH)	[4,5]
13	PF3D7_1349200	glutamate--tRNA ligase, putative	[11]
14	PF3D7_1407900	plasmepsin I (PMI)	[12]
15	PF3D7_0525100	acyl-CoA synthetase (ACS10)	[5]
16	PF3D7_1407800	plasmepsin IV (PM4)	[12]
17	PF3D7_0624000	hexokinase (HK)	[5,13]
18	PF3D7_0709000	chloroquine resistance transporter (CRT)	[3,10]
19	PF3D7_1332900	isoleucine-tRNA ligase, putative	[14]
20	PF3D7_0629200	DnaJ protein, putative	[2]
21	PF3D7_0322000	peptidyl-prolyl cis-trans isomerase (CYP19A)	[5]
22	PF3D7_1408000	plasmepsin II	[12]
23	PF3D7_1029600	adenosine deaminase, putative	[4–6]
24	PF3D7_0513300	purine nucleoside phosphorylase (PNP)	[4,6,7]
25	PF3D7_1034900	methionine-tRNA ligase, putative	[15]
26	PF3D7_0823800	DnaJ protein, putative	[2]
27	PF3D7_1308200	carbamoyl phosphate synthetase (cpsSII)	[6,7]
28	PF3D7_1344800	aspartate carbamoyltransferase (atcasE)	[4–6]
29	PF3D7_0209800	ATP-dependent RNA helicase UAP56 (UAP56)	[2,5]
30	PF3D7_0621200	pyridoxine biosynthesis protein PDX1 (PDX1)	[6]
31	PF3D7_1416500	NADP-specific glutamate dehydrogenase (GDHa)	[5]
32	PF3D7_1408100	plasmepsin III,histo-aspartic protease (HAP)	[12]
33	PF3D7_0709700	lysophospholipase, putative	[7]

Supplementary Table 3 | The predicted peptide fragments of TCTP after *in silico* trypsin digestion.

Position of cleavage site	Resulting peptide sequence*	Peptide length [aa]	Peptide mass [Da]	Cleavage probability
2	MK	2	277.382	100 %
5	VFK	3	392.499	87.9 %
30	DVFTNDEVCSDSYVQQD PFEVPEFR	25	2966.139	69.6 %
37	EIAFEVK	7	834.968	100 %
40	SNK	3	347.371	65.1 %
41	R	1	174.203	83.4 %
43	IK	2	259.349	100 %
84	GNEDYGIADNSEDAVEG MGADVEHVIDIVDSFQLTS TAFSK	41	4347.598	84.2 %
85	K	1	146.189	84.6 %
92	EYSAYIK	7	872.973	89.7 %
97	NYMQK	5	682.792	100 %
100	VAK	3	316.401	100 %
105	YLEEK	5	680.756	87.3 %
106	K	1	146.189	44.5 %
109	PDR	3	386.408	100 %
114	VEIFK	5	634.773	100 %
116	TK	2	247.294	100 %
122	AQPFIK	6	702.852	100 %
151	HILTNFDDFEFYMGESLD MEAGIIYSYYK	29	3512.907	88.2 %
154	GEE	3	333.298	-

*Most of the peptides are either too long or two short, thus the chance to identify TCTP in a mixture of trypsin-digested proteins is low (only the two peptides highlighted in red are more prone to MS/MS identification).

Supplementary Table 4 | The IC_{50} values of artesunate and artesunate in the presence of a haem modulator (ALA, SA). The artesunate and haem modulator were co-incubated with the parasite culture for the whole culturing course. Data are mean ± s.d.; n=3.

	Artesunate	Artesunate + ALA	Artesunate + SA
IC_{50} (nM)	18.8 ± 1.6	17.9 ± 1.7	17.4 ± 1.3

Supplementary Table 5 | Oligonucleotide primers used for cloning.

Gene	NCBI reference sequence		Sequence[a]	Restriction enzymes for cloning	Tag
**LDH**	XM_001349953	Sense	CGTTCCATGG**CACCAAAAGCAAAAATCG**	Nco I/Xho I	C-6xHis
		Antisense	CGTTCTCGAG**AGCTAATGCCTTCATTCTCTTAG**		
**OAT**	XM_960985	Sense	CATGCC**ATGGATTTCGTTAAAGAATTAA**	Nco I/Xho I	C-6xHis
		Antisense	CATGCTCGAG**TAAATTGTCATCAAAAAATTTAAC**		
**PyrK[b]**	XM_961158	Sense	CTACTAGCTAGC**GCGGCCGGCGCAAGTATG**	Nhe I/Xho I	N-6xHis
		Antisense	CATGCTCGAG**TCACTCAATTTGTACCACCTTCATCAGGTTG**		
**SAMS[c]**	XM_001352057	Sense	CGTTCCATGGCA**AGTCAGTTGAAAATTAAAAGAG**	Nco I/Xho I	C-6xHis
		Antisense	CGTTCTCGAG**ATTTTTTAATGCATTTTTTTC**		
**SpdSyn**	XM_001347936	Sense	CGTTCCATGGCA**GATAAACTGATTAGCAATAACAAG**	Nco I/Xho I	C-6xHis
		Antisense	CGTTCTCGAG**GATGTTCTCGATCTCTTTCAAC**		
**TCTP**	XM_001351631	Sense	CTACTAGCTAGC**ATGAAAGTATTTAAAGACGTT**	Nhe I/Xho I	N-6xHis
		Antisense	CATGCTCGAG**TTAATATTTTTCTTCAAAAAGTC**		

[a]The restriction enzyme sites for cloning are underlined and the genes of *P. falciparum* are bolded.

[b]The gene is codon optimized and without first 10 amino acids.

[c]The gene is codon optimized.

Supplementary References

1. Berger, B. J. Antimalarial activities of aminooxy compounds. *Antimicrobial Agents and Chemotherapy* **44,** 2540–2542 (2000).
2. Ludin, P., Woodcroft, B., Ralph, S. a & Mäser, P. In silico prediction of antimalarial drug target candidates. *International Journal for Parasitology: Drugs and Drug Resistance* **2,** 191–199 (2012).
3. Ding, X. C., Beck, H. P. & Raso, G. Plasmodium sensitivity to artemisinins: Magic bullets hit elusive targets. *Trends in Parasitology* **27,** 73–81 (2011).
4. Huthmacher, C., Hoppe, A., Bulik, S. & Holzhütter, H.-G. Antimalarial drug targets in Plasmodium falciparum predicted by stage-specific metabolic network analysis. *BMC systems biology* **4,** 120 (2010).
5. Crowther, G. J. *et al.* Identification of attractive drug targets in neglected- disease pathogens using an in Silico approach. *PLoS Neglected Tropical Diseases* **4,** e804. (2010).
6. Plata, G., Hsiao, T.-L., Olszewski, K. L., Llinás, M. & Vitkup, D. Reconstruction and flux-balance analysis of the Plasmodium falciparum metabolic network. *Molecular systems biology* **6,** 408 (2010).
7. Yeh, I., Hanekamp, T., Tsoka, S., Karp, P. D. & Altman, R. B. Computational analysis of Plasmodium falciparum metabolism: organizing genomic information to facilitate drug discovery. *Genome Research* **14,** 917–924 (2004).
8. Van Brumelen, A. C. *et al.* Co-inhibition of Plasmodium falciparum S-Adenosylmethionine decarboxylase/ornithine decarboxylase reveals perturbation-specific compensatory mechanisms by transcriptome, proteome, and metabolome analyses. *Journal of Biological Chemistry* **284,** 4635–4646 (2009).
9. Eckstein-Ludwig, U. *et al.* Artemisinins target the SERCA of Plasmodium falciparum. *Nature* **424,** 957–961 (2003).
10. Müller, I. B. & Hyde, J. E. Antimalarial drugs: modes of action and mechanisms of parasite resistance. *Future microbiology* **5,** 1857–1873 (2010).
11. Jain, V., Kikuchi, H. & Oshima, Y. Structural and functional analysis of the anti-malarial drug target prolyl-tRNA synthetase. *Journal of Structural and Functional Genomics* **15,** 181–190 (2014).
12. Rosenthal, P. J. Proteases of malaria parasites: New targets for chemotherapy. *Emerging Infectious Diseases* **4,** 49–57 (1998).
13. Fatumo, S. *et al.* Estimating novel potential drug targets of Plasmodium falciparum by analysing the metabolic network of knock-out strains in silico. *Infection, Genetics and Evolution* **9,** 351–358 (2009).
14. Pham, J. S. *et al.* Aminoacyl-tRNA synthetases as drug targets in eukaryotic parasites. *International Journal for Parasitology: Drugs and Drug Resistance* **4,** 1–13 (2014).

15. Jackson, K. E. *et al.* Dual targeting of aminoacyl-tRNA synthetases to the apicoplast and cytosol in Plasmodium falciparum. *International Journal for Parasitology* **42,** 177–186 (2012).
